# Matching Low Viscosity with Enhanced Conductivity in Vat Photopolymerization 3D Printing: Disparity in the Electric and Rheological Percolation Thresholds of Carbon-Based Nanofillers Is Controlled by the Matrix Type and Filler Dispersion

**DOI:** 10.1021/acsomega.3c05683

**Published:** 2023-11-25

**Authors:** Veronika Sevriugina, David Pavliňák, František Ondreáš, Ondřej Jašek, Martina Štaffová, Petr Lepcio

**Affiliations:** † Central European Institute of Technology, Brno University of Technology, Purkyňova 656/123, 612 00 Brno, Czech Republic; ‡ Contipro a.s., Dolní Dobrouč 401, 561 02 Dolní Dobrouč, Czech Republic; § Department of Physical Electronics, Faculty of Science, Masaryk University, Kotlářská 267/2, 611 37 Brno, Czech Republic

## Abstract

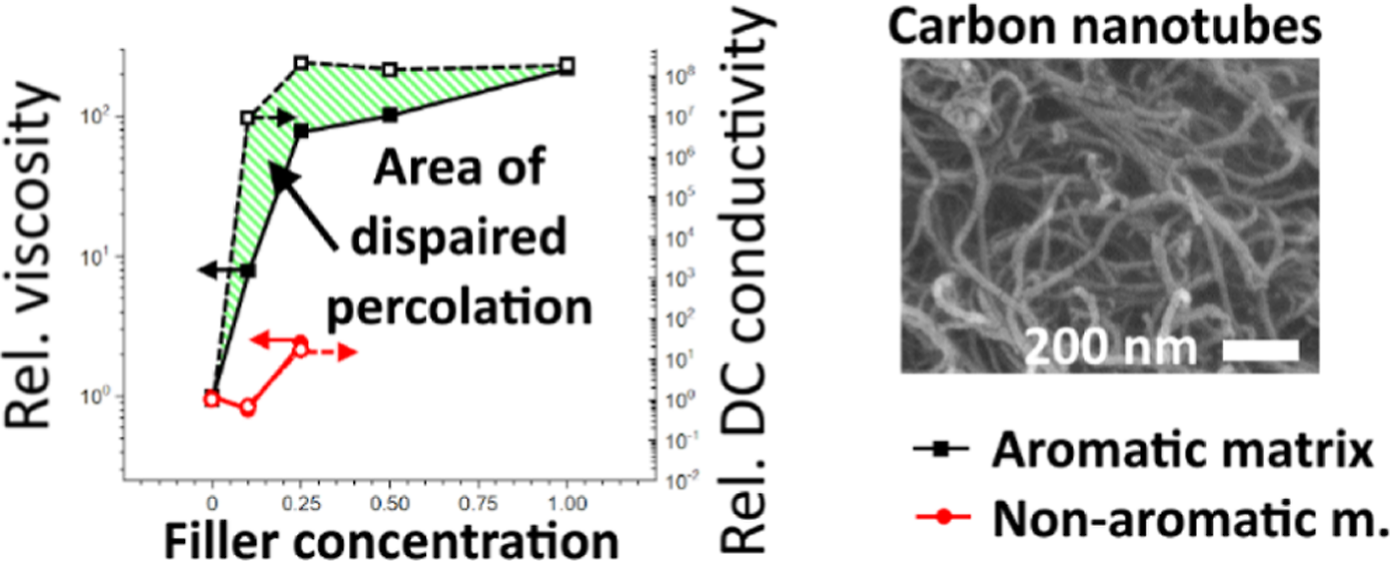

This study investigated the impact of carbonaceous fillers (carbon black, multiwalled carbon nanotubes, graphene, and highly defective graphene) on aromatic and nonaromatic photopolymer resins’ properties, such as viscosity, long-term stability, complex permittivity, curing efficiency, final conversion, storage modulus, heat deflection and glass transition temperatures, network density, and DC resistivity. The presented results also highlight challenges that must be addressed in designing and processing carbonaceous filler-based 3D-printed photopolymer resins. The improved dielectric and electrical properties were closely tied to the dispersion quality and filler–matrix affinity. It favored the enhanced dispersion of anisotropic fillers (nanotubes) in a compatible matrix above their percolation threshold. On the other hand, the dispersed filler worsens printability due to the elevated viscosity and deteriorated penetration depth. Nonetheless, electrical and rheological percolation was found at different filler concentrations. This window of despaired percolation combines highly enhanced conductivity with only mildly increased viscosity and good printability.

## 1 Introduction

Nanoparticles (NPs) and other nanosized fillers can enhance or add new properties to the embedding polymer matrix.^1−3^ Among them, carbonaceous nanomaterials such as carbon black (CB), carbon nanotubes (CNTs), or graphene nanoplatelets (GNPs) are favored for improving dielectric, electrical, thermal, and mechanical characteristics.^4−6^ The potential applications involve electromagnetic (EM) interference shielding, electrostatic dissipation,^7^ electronic circuits, sensors, and transparent flexible electrodes for displays and solar cells.^8^ Carbonaceous nanofillers increase the nanocomposite’s electrical conductivity by changing its complex permittivity. Most carbon-based materials are electrically and thermally conductive due to the delocalized π electrons (sp^2^).^9^ The exception is diamond-like structures with prevailing sp^3^ hybridization.^10^

The conductivity values reported in the literature for various systems, including thermoplastics and photopolymers, typically range from ∼10^–7^ to 10^–1^ S·cm^–1^.^11−13^ Cross-linking exfoliated graphene with metal cations in 1:1 w/w graphene/nanocellulose films recently reported an enhancement of up to ∼10^1^ S·cm^–1^, boosting the EM shielding effectiveness (SE) up to nearly 800 dB·mm^–1^ in the microwave range.^14^ In contrast, bulk nanocomposites reach values up to ∼50 dB·mm^–1^ at 6 vol % of the filler when utilizing the synergistic effect of CNTs/CB or CNTs/GNPs.^14^ Several studies also found exceptionally high dielectric constant values near the percolation threshold.^15−19^

Nonetheless, the reported results for improved conductivity, thermal stability, and mechanical properties are disparate. The optimal filler concentration varied across a broad range between 0.5 and 25%.^11−13,15−20^ The reason is not only the various types, shapes, and sizes of fillers but also mainly the chemical nature of the polymer and the dispersion quality.^7,21^ The distribution of nanofillers into the polymer matrix (homogeneous dispersion, agglomerated, or clustered) critically affects the behavior and properties of nanocomposites.^22,23^ Favorable polymer–filler interactions help achieve better dispersion, reducing the percolation concentration. For instance, CNTs in isotactic polypropylene percolated at 1.24 vol %, while the percolation threshold was only 0.33 vol % in polycaprolactone.^24^ Partial aggregation shifts the percolation threshold to a higher concentration, but the percolated aggregates show higher EM shielding effectivity than a nonaggregated filler.^25,26^ Furthermore, different properties may display different percolation thresholds. Huang et al. reported close percolation concentrations for melt viscosity and electric conductivity, but 4–6 times higher values for EM shielding.^24^

Black carbonaceous fillers absorb light in a broad spectral range. Thus, they undesirably change a photopolymer resin’s optical clarity and penetration depth unless a fine dispersion of small NPs (<100 nm) is maintained well below the light’s wavelength.^8^ Any larger structures would interact with the curing light and reduce its effective intensity available for photoinitiation.^4,6,27^ Some nanofillers may also provide additional contributions such as the photothermal effect or shift of the photoinitiator redox potentials.^28^ That is probably why studies employing conductive carbonaceous fillers in vat photopolymerization (VPP) are far less common than the analogical reports on material extrusion techniques.

Filler’s spatial and orientational distribution in a liquid medium could be quantitatively correlated to the rheological properties.^22,29^ Monomer adsorption on the nanofiller’s surface often increases the resin’s viscosity by enlarging the filler’s effective hydrodynamic volume. This monomer immobilization may lower the critical curing dose needed to form a solid layer and worsen the resin’s spreadability.^28^ The effect is especially pronounced above the percolation threshold, where a network of physically interconnected fillers is created. This represents a favored state for achieving good conductivity because the continuous conductive path eases the charge transfer through the material. However, it may easily turn a liquid resin into a soft paste-like solid or a gel with a limited capability to print fine details.^4,30,31^

The current work comprehensively compares viscosity, dispersion stability, photocurability, and final thermomechanical and dielectric properties regarding the filler’s shape, concentration, dispersion, and affinity to the polymer matrix. It investigates these parameters using CB, CNTs, and graphene in commercial aromatic and nonaromatic photocurable acrylic resins. It identifies parameters critical for promoting electrical conductivity and permittivity and correlates them to downgraded processability. Interestingly, unlike the study of Huang et al.,^24^ it reveals a disparity in the electric and rheological percolation thresholds, opening a window for low-viscosity resins with enhanced electrical performance. These findings are of utmost practical interest to all workers employing carbon-based nanofillers in photocurable resins, not only for additive manufacturing but also for other photopolymerization processes. Hence, the reported results guide a potential user through all essential steps, including selection, preparation, processing, and characterization of the photopolymerized carbonaceous nanocomposites.

## 2 Materials and Methods

### 2.1 Materials

The carbonaceous materials (CB, CNTs, and graphene) were selected as representative 0D, 1D, and 2D nanofillers, respectively. Highly electroconductive CB Chezacarb prepared by gasification of heavy petroleum residues using autothermic noncatalytic partial oxidation was kindly provided by ORLEN Unipetrol (Czech Republic). It was obtained in 0.5–2.5 mm pellets of tightly aggregated 60 nm primary particles. Nanocyl, S.A (Belgium) provided multiwalled CNTs (MWCNTs) NC7000. They were manufactured by a catalytic chemical vapor deposition process and purified to an average carbon purity of 90%. The average diameter of a nanotube is 5 nm, and its average length is 1.5 μm. Graphene containing 1–1.5% oxygen prepared by a “bottom-up” laboratory-scale method using a microwave atmospheric plasma torch^32^ was kindly provided by Masaryk University (Czech Republic). The oxygen content in highly defective graphene (h-d graphene) was increased to 2–3%. The Brunauer–Emmett–Teller (BET) (3P Micro 300C1, 3P Instruments, Germany), X-ray photoelectron spectroscopy (XPS) (Axis Supra, Kratos, UK), Raman spectroscopy (WITec alpha300 R, Oxford Instruments, UK), and scanning electron microscopy energy-dispersive X-ray spectroscopy (SEM–EDX) (Mira 3 XMU, Tescan, Czech Republic with X-Max 20 EDX detector, Oxford Instruments, UK) analyses of all fillers are provided in the Supporting Information (Figure S1–S3 and Table S1–S3). A commercial acrylic Hard transparent 3D printing resin (Shenzhen Yongchanghe Technology, China) containing 6% of the diphenyl(2,4,6-trimethylbenzoyl)phosphine oxide photoinitiator was used as an aromatic matrix. The nonaromatic resin was prepared by mixing SR833S (tricyclodecanedimethanol diacrylate), SR9003 (propoxylated 2 neopentyl glycol diacrylate), and CN966H90 (aliphatic urethane acrylate diluted with 10% of 2-(2-ethoxyethoxy)ethyl acrylate) monomers (all ARKEMA Sartomer, NL) in the ratio of 3:3:4 with 3% of the phenylbis(2,4,6-trimethylbenzoyl)phosphine oxide photoinitiator (RAHN, Switzerland) at 60 °C for 1 h. The aromatic character is evidenced by double-bond triplets in the Fourier transform infrared (FTIR) spectra (Figure S4a–c), which are absent in the nonaromatic resin (Figure S4d,e).^33^

### 2.2 Sample Preparation

Samples were prepared by mixing the nanofillers into the photopolymer resin at different concentrations and stirring with a magnetic stirrer (IKA RCT Basic) for 10 min. Samples marked as “dispersed” were sonicated using an ultrasonic homogenizer (Bandelin Sonopuls HD 3200) for 3 min. The formulations were 3D printed with an Original Prusa SL1 VPP 3D printer equipped with an LED light source (VPP-LED). The wavelength and light intensity were 405 nm and 0.661 mW·cm^–2^, respectively. The layer thickness was 50/25 μm for aromatic/nonaromatic matrix samples. Unless otherwise stated, the first layer was exposed for 120 s and all others were exposed for 30 s. The printing instructions were generated in the OEM software Prusa Slicer (v. 2.4.0).

### 2.3 Jacobs Working Curves

Creating the Jacobs working curve has become essential for optimizing the 3D printing process of new photocurable systems through stereolithography (SLA) or digital light processing (DLP).^34^ It provides information about the dependence of the cure depth (*C*_d_) on the exposure energy (*E*_0_), the product of the light intensity (*I*_0_), and the curing time (*t*). The intersection of the Jacobs working curve with the *x*-axis represents the critical energy (*E*_c_) required to initiate polymerization, and the slope of the curve represents the penetration depth (*D*_p_) (see eq 1).^35,36^
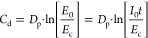
1

Single-layer square 10 × 10 mm samples were cured without a printing platform while varying the exposure time from 10 to 300 s. Jacobs working curves were created by plotting the thickness of the cured layer against the exposure energy logarithm.

### 2.4 Rheology

Rheological properties were assessed with an advanced rotational rheometer DHR-2 (TA Instruments, USA) at 30 °C isothermal conditions, using a 40 mm parallel plate geometry and a gap of 1 mm. Oscillatory frequency and strain amplitude tests were performed. According to the empirical Cox–Merz rule, the steady-state viscosity’s shear rate dependence equals the complex viscosity’s angular frequency dependence. The power law index was evaluated from the complex viscosity obtained in a frequency sweep test ranging from 0.1 to 20 Hz at a strain of 0.1%. The power law index *n* signals the viscosity depression in the shear-thinning power law region (eq 2)
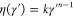
2

The variables η, γ′, and *k* represent the viscosity, shear rate, and consistency, respectively.^37^ On top of that, the 1 Hz linear viscoelastic region (LVR) complex viscosity η_1 Hz_ was determined from the linear part of the strain amplitude sweep test performed at the strain amplitude ranging between 0.1 and 50% and the reference frequency of 1 Hz.

### 2.5 FTIR Spectroscopy

FTIR spectra were collected using a Vertex 70 V vacuum spectrometer (Bruker, Germany) with an attenuated total reflectance (ATR) module. The analysis was carried out in the spectral range of 4000–600 cm^–1^ with 1 cm^–1^ resolution and 32 scans. A background measurement was run before each sample’s spectrum collection. The FTIR spectra were normalized to the C=O group peak area at 1730 cm^–1^. The double bond conversion was calculated by integrating the peak area of the C=C group in the 1650–1600 cm^–1^ region. The aromatic resin contained nonreactive aromatic double bonds contributing 80.94% of the total double-bond signal (Figure S4c).^28^ Therefore, the conversion was determined from the peak found at 1650–1600 cm^–1^ after deconvolution. Due to the single-sided illumination setup used in the 3D printer, we measured both sides of a 1 mm thick 3D printed specimen. The side marked as the upper represents the first layer attached to the aluminum build platform, while the bottom side corresponds to the last layer abutted to the LED light source.

### 2.6 Hybrid Dynamic Mechanical Analysis Combined with Heat Deflection Temperature Measurement

Hybrid dynamic mechanical analysis (DMA) combined with heat deflection temperature (HDT) measurement, a method well-suited for characterizing 3D printed materials,^34,38^ was performed using RSA-G2 (TA Instruments, USA) on (3.5 × 5 × 48.5) mm^3^ specimens. The analysis was carried out in the 3-point bending setup at a controlled heating rate of 2 °C·min^–1^ from 30 to 160 °C, an oscillatory frequency of 1 Hz, and a dynamic oscillation strain of 0.002%. The stiffness was represented by storage modulus (*G*′) and evaluated at 35 °C in the glassy plateau region. The maximum tan δ peak denoted the glass transition temperature (*T*_g_). The HDT was established as the temperature at which the flexural strain reached 0.195%, corresponding to the normalized body’s deflection of 0.25 mm. The flexural strain (ε_f_) was calculated according to eq 3.^34^
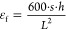
3where *s* is the deflection, *h* is the thickness, and *L* is the span.^34^ The network density (*v*_e_) was calculated using the storage modulus value in the Rubbery plateau region (*G*_*N*_^0^) determined at 95 and 140 °C for composites based on aromatic and nonaromatic matrixes, respectively (eq 4).
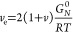
4where *v* is the Poisson’s ratio (*v* = 0.5 for incompressible material), *R* is the gas constant, and *T* is the temperature.^28^

### 2.7 Tensile Test

The mechanical properties in tension were measured at a rate of 2 mm·min^–1^ on dumbbell-shaped bodies with a 10 mm gauge length by the Zwick Roell Z010 (Zwick-Roell, Germany) testing machine equipped with a 1 kN force sensor. The test ended at the test specimen’s break, which was identified by a drop of the maximum force by 50%.

### 2.8 Dielectric Thermal Analysis

Dielectric properties were investigated by the ARES-G2 rheometer (TA Instruments, USA) equipped with an OEM dielectric accessory. It consists of 25 mm parallel plates with built-in electrodes and ceramic insulation, using an Agilent E4980A precision LCR meter for field control and measurement. Data were collected from 3D printed 25 × 1 mm discs and uncured liquid resins to monitor the dielectric permittivity and its change over time. The zero time was set as equal to the ultrasonication step. The oscillating voltage signal of 1 V was used at frequencies from 20 Hz to 2 MHz under isothermal conditions at 30 °C. In addition, an axial force of 1 N was applied to the printed disks to obtain better contact between the solid specimens and the geometries.

The determined frequency-dependent complex dielectric permittivity ε* consists of the real part, the relative dielectric constant ε′, and the imaginary part, the dielectric loss factor ε″ (see eq 5).^39^
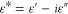
5where *i* is the imaginary number. The dielectric loss factor tan δ was also obtained as a function of frequency given by eq 6.^39^
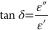
6

### 2.9 DC Electrical Resistance

The DC volume and surface resistivity of printed square specimens (40 × 40 × 1 mm) were measured according to ASTM D257. The measurement was performed by Fisher-Elektronik Milli-TO 3 (Fisher Elektronik, Germany) using a shielded electrode FE-25 with an outer diameter of 40 mm, a bipolar electrode connection, and an electrification time of 1 min. The analysis was carried out at a voltage of 500 V, a temperature of 22 °C, and a relative humidity of 70%. Conductive rubbers were used to obtain better adhesion of the samples to electrodes. Due to the photosensitivity of the tested materials, the samples were stored in a dark box before the measurements to avoid arbitrary curing by ambient light.

### 2.10 Scanning Electron Microscopy

The SEM characterized the CNT dispersion using a Mira 3 XMU (Tescan, Czechia) at 30 kV acceleration voltage. Specimens were coated with a 12 nm gold layer sputtered by an EM ACE 600 (Leica, Germany). The images were collected by a standard Everhart–Thornley secondary electron detector from the top side of the photopolymer resins cured by a 405 nm LED without a printing platform.

## 3 Results and Discussion

### 3.1 Rheological Properties

Two commercial photopolymer systems were selected to test the effect of the matrix–filler affinity. The aromatic matrix has a good affinity to the carbonaceous fillers, facilitated by its moieties with sp^2^ hybridization. Moreover, the delocalized electrons contribute to its electrical and dielectric properties, as will be discussed later. On the other hand, the nonaromatic resin lacks the characteristic double-bond triplet in the FTIR spectra (Figure S4d,e), evidencing the absence of the aromatic cycles that can strongly interact with the carbonaceous fillers and provide delocalized electrons.^33^

**Figure 1:**
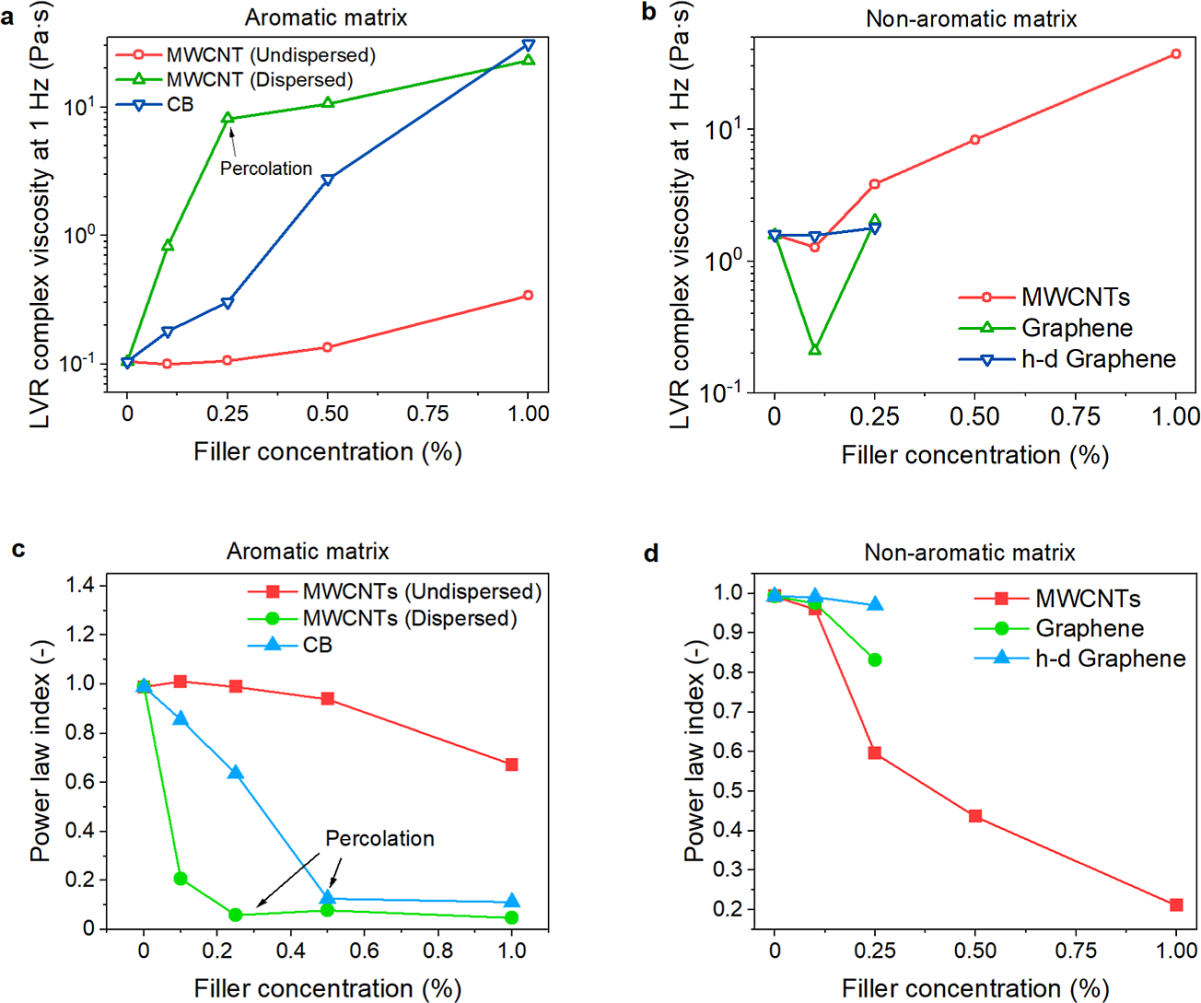
(a,b) LVR complex viscosity at 1 Hz (η_1 Hz_) and (c,d) power law index *n* as functions of the filler concentration for the (a,c) aromatic and (b,d) nonaromatic matrix nanocomposite resins.

Resin’s viscosity is a vital characteristic determining the material’s spreadability. Rheological properties also provide indirect evidence of the filler distribution and its interaction with the matrix.^22^ The graphs in Figure S5a (aromatic resin) and S5b (nonaromatic resin) show the complex viscosity as a function of angular frequency at 30 °C. Notably, the filler’s impact on viscosity is more pronounced at low frequencies,^40^ giving it a higher sensitivity to structural changes. Therefore, the viscosity (η_1 Hz_) dependence on the filler’s concentration is plotted in Figure 1a,b at a representative oscillation frequency of 1 Hz (≈6.28 rad·s^–1^). On top of that, the viscosity increase was accompanied by the typical shear-thinning behavior characterized by decreasing complex viscosity at higher angular frequencies (Figure S5a). This so-called power law region is described with a power law index *n* (eq 2), which indicates the strength of intermolecular interactions (Figure 1c,d and Table S5).^37^

Nanofillers often increase the viscosity of polymer liquids to an extent that has not been seen for analogous microfillers due to the monomer/polymer adsorption on the nanofillers’ large surface area. That increases the effective hydrodynamic volume and, in turn, the viscous drag experienced by the free-flowing liquid in a shear field.^22^ Such a behavior denoted by the elevated viscosity (Figure 1a) was observed in the filled aromatic matrix resins, supporting the expectation of a good affinity between the aromatic matrix and the carbonaceous fillers.^22^ Nonetheless, the trend varied with the filler type (CB and MWCNTs) and dispersion quality (dispersed and undispersed MWCNTs).

The η_1 Hz_ increased by 2 times for the sample with 1% of undispersed MWCNTs, but it grew by over 2 orders of magnitude for the same amount of dispersed MWCNTs or CB (Figure 1a). Dry-state nanofillers are aggregated due to the attractive van der Waals forces, and their redispersion requires an extensive shear force.^22^ Polymer solutions and other low-viscosity liquids, such as photopolymer resins, are usually treated with high-energy ultrasonic probes generating extreme local shear conditions due to the cavitation.^22^ Freshly dispersed filler must be stabilized to prevent reaggregation, either by electrostatic repulsion if the zeta potential is larger than ±10 mV, or by steric repulsion facilitated by the adsorbed monomer/polymer.^41^ While the well-dispersed filler is dominated by the matrix–filler interaction, the filler–filler interaction prevails in aggregates, leaving only a tiny portion of the filler’s surface area for interaction with the polymer matrix. We have previously demonstrated that the nanofiller’s dispersion state correlates with the matrix–filler interaction strength, viscosity in a solution^22^ and the melt, reptation time, glass transition temperature, yield stress, storage modulus, and other thermomechanical properties.^42^ The correlation between dispersion and photocuring, electrical, and dielectric properties can be found in this study.

The improved dispersion caused by ultrasonication turned the formulations into a soft gel or viscous paste due to the formation of filler superstructures, which can support a mechanical load.^29^ Such materials are conveniently printed at elevated temperatures, reducing their high viscosity.^43^ The percolation threshold, marked by an abrupt increase of η_1 Hz_, was observed already at 0.25 and 0.5% of dispersed MWCNTs and CB, respectively. Indeed, anisotropic nanotubes are expected to percolate at a lower concentration than the more isotropic CB.^14,44^ The low *n* values also indicate that the MWCNTs formed a more extended network in the aromatic resin than in CB (Figure 1c and Table S5). Notably, *n* flats out to nearly constant values for dispersed MWCNTs (≈0.06) and CB (≈0.12) above their percolation thresholds. The *n* remains high (>0.67) for the undispersed MWCNTs though (Figure 1c), corresponding with no hints of percolation in Figure 1a.

Figures 1b,d and S5b,d show analogical rheological data for the samples with the nonaromatic matrix. The complex viscosity of most mixtures was almost frequency independent (Figure S5b) with a power law index *n* close to 1 (Figure 1d and Table S5), indicating Newtonian behavior. However, the 0.25, 0.5, and 1% MWCNT samples showed *n* = 0.596, 0.437, and 0.211, respectively. Nonetheless, these values are 1.5–2 orders of magnitude greater than in the aromatic resin (0.059, Figure 1c and Table S5), marking the much less extended MWCNT network due to the weak interactions with the nonaromatic resin. Although the nonaromatic resin had a higher viscosity than the aromatic one, the filled formulations remained liquid up to 1% of MWCNTs. Interestingly, 0.1% of MWCNTs and graphene reduced the viscosity by 19.4 and 86.7%, respectively (Figure 1b and Table S5). Such an effect is attributed to the depletion attraction, suggesting an exceptionally weak interaction between the filler and the matrix.^22^ On the other hand, no viscosity drop in 0.1% of highly defective graphene testifies to the enhanced attraction of the more hydrophilic filler (see the atomic composition in Table S2). The η_1 Hz_ (Figure 1b) revealed the percolation thresholds of MWCNTs and graphene composites to be near 0.25 and 0.1%, respectively. However, highly defective graphene nanocomposites did not exhibit such a transition up to 0.25% and could not be processed at higher concentrations.

Additionally, we tested the dispersion stability by repeating the rheological measurement over time (1, 14, and 28 days after ultrasonication). The nanosuspensions obtained by ultrasonication were mainly stable within the tested time frame, as confirmed by the η_1 Hz_ time function (see Figure S5c,d). It ensures constant properties through 3D printing and potentially even long-term storage. The only hints of aggregation testified by lowered viscosity appeared in the 0.25% MWCNTs/nonaromatic resin sample after 2 weeks of storage (Figure S5d).

### 3.2 Electrical/Dielectric Properties of Uncured Resins

**Figure 2:**
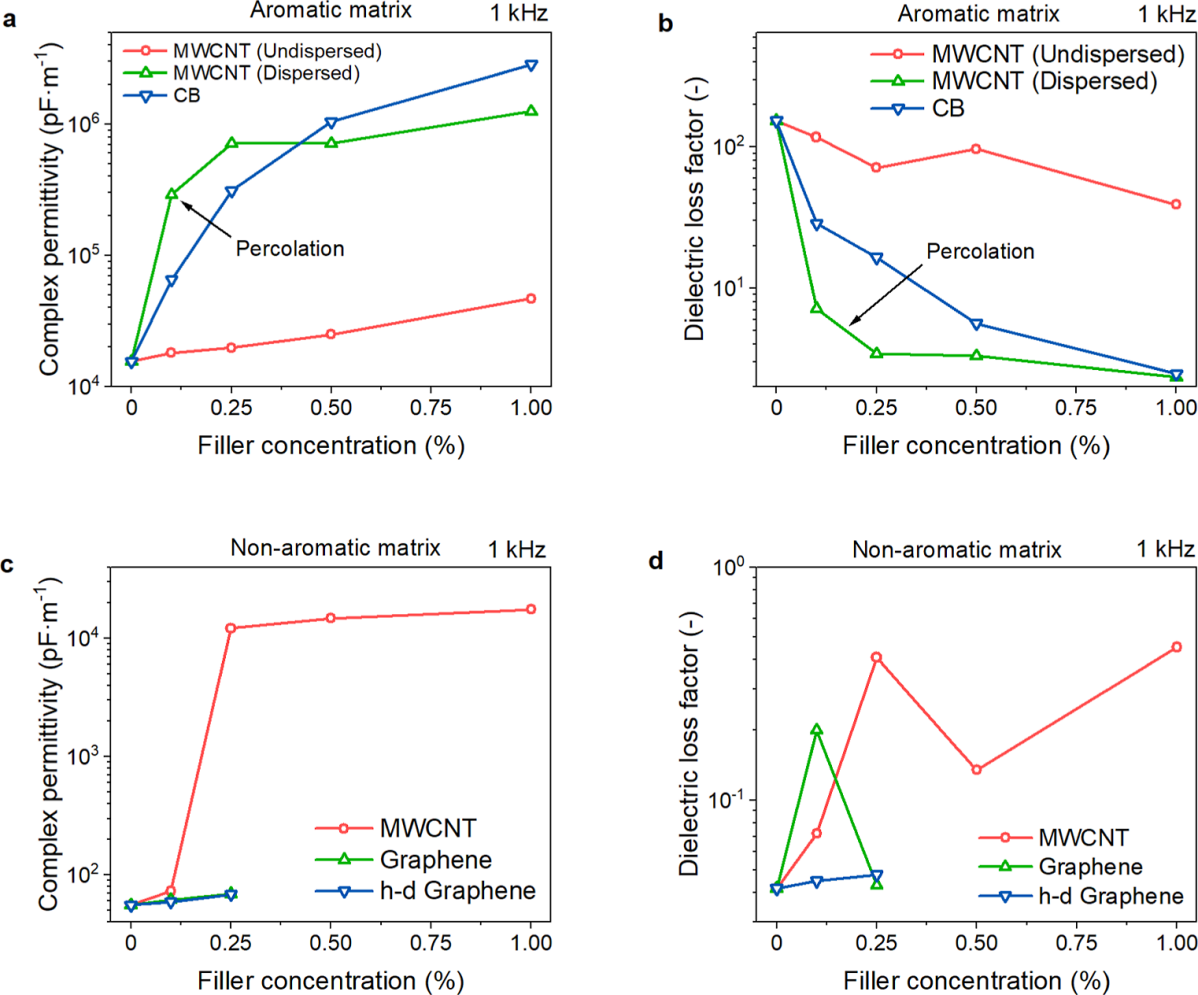
(a,c) Complex permittivity and (b, d) dielectric loss factor at 1 kHz as functions of the filler concentration for the uncured (a, b) aromatic and (c,d) nonaromatic matrix nanocomposite resins.

Dielectric thermal analysis (DETA) was performed on uncured resins to establish the dielectric percolation threshold. Polarization is the crucial dielectric property stemming from the alignment of charged species under an external electric field. It comes from four sources: electronic, ionic, orientational, and interfacial polarization. The active modes of polarization depend on the operation frequency.^39^ All of the frequency data of aromatic matrix mixtures’ complex permittivity are available in Figure S6a. The frequency data showed a typical decreasing tendency since the slow polarization mechanisms could not fully contribute at high frequencies. Figure 2a,b shows the complex permittivity and loss tangent values for the aromatic matrix resins as a function of filler’s content at a representative frequency of 1 kHz at 30 °C. Notably, the complex permittivity of undispersed MWCNTs formulations steadily increased with concentration, even though no percolation threshold was detected (Figure 2a). On the contrary, ultrasonication increased the permittivity at 1 kHz by 2 orders of magnitude through the enhanced dispersion. The lower impact of undispersed MWCNTs can be explained by large polarizable aggregates separated by vast regions of the low-permittivity medium.

A trend change in the loss tangent, a property describing dielectric losses, is observed above 0.25% for undispersed MWCNTs (Figure 2b). Nonetheless, the loss tangent values remain much higher than for the dispersed MWCNTs or CB. The dielectric percolation threshold appeared between 0.1 and 0.25% for CB and dispersed MWCNTs according to the loss tangent (Figure 2b). Moreover, CB yielded a less abrupt change in permittivity than dispersed MWCNTs (Figure 2a), analogously to the rheological results (Figure 1a). It suggests that the CB percolation has a more diffuse character than the MWCNTs. Nonetheless, the electric percolation (Figure 2a,b) appeared at a lower concentration than the rheological one (Figure 1a,c). A possible explanation is quantum mechanical tunneling when the neighboring NPs are in near contact. This way, electrons can bridge short distances across the insulating matrix.^45^ This disparity opens an opportunity for matching low-viscosity resins with enhanced electrical properties.

Time-dependent DETA measurements of ultrasonicated nanocomposites (Figure S6b) further support the rheological observation. Stable uniform MWCNT dispersions lasted for at least 28 days after preparation. Some hints of aggregation were observed only for 1% of MWCNTs, while increased complex permittivity was observed in the 0.25% MWCNTs sample. On the contrary, CB dispersion stability was found to be worse for low concentrations and stable at higher loadings.

The dielectric properties of nonaromatic resins are given in Figure 2c,d, and Table S6. The filling had no significant influence on the dielectric properties. Slight differences were apparent only in the low-frequency region. The only significant change is noticeable for the 0.25% of MWCNTs sample at 1 kHz, where the complex permittivity (1.22 × 10^4^ pF·m^–1^) increased by 2 orders of magnitude. The dielectric percolation threshold was determined from the complex permittivity (Figure 2c) and loss factor values (Figure 2d) as functions of the filler content at 1 kHz and 30 °C. Similar to the rheological measurements, the percolation thresholds for MWCNTs and graphene in a nonaromatic matrix were established near 0.25 and 0.1%, respectively. Highly defective graphene caused no sharp changes in the dielectric properties. The mixtures were found stable over 28 days, with only minor changes in the 0.25% MWCNTs sample after 14 days (Figure S6d). Interestingly, the tan δ of this sample was 0.41, i.e., 8.3 times lower than that in the aromatic matrix with the same filling (3.41). Therefore, a nonaromatic matrix will dissipate less energy in AC electric fields.

Comparing the results obtained for aromatic and nonaromatic matrices with the study of Huang et al.^24^ suggests that the despaired electric and rheological percolation is allowed by the specific electronic properties of the embedding matrix. The disparity was observed only in the aromatic matrix featuring the sp^2^ hybridized aromatic rings with delocalized π electrons. The analogical structure is responsible for the high conductivity of carbonaceous fillers.^9^ Simulations predicted the tunneling current dependence on a covalently bonded molecule’s bonding sites and angles on a disordered sp^2^ hybridized carbon surface, suggesting that electron tunneling favors mediation by orbitals with significant electron density.^46^

### 3.3 Curing Properties

**Figure 3:**
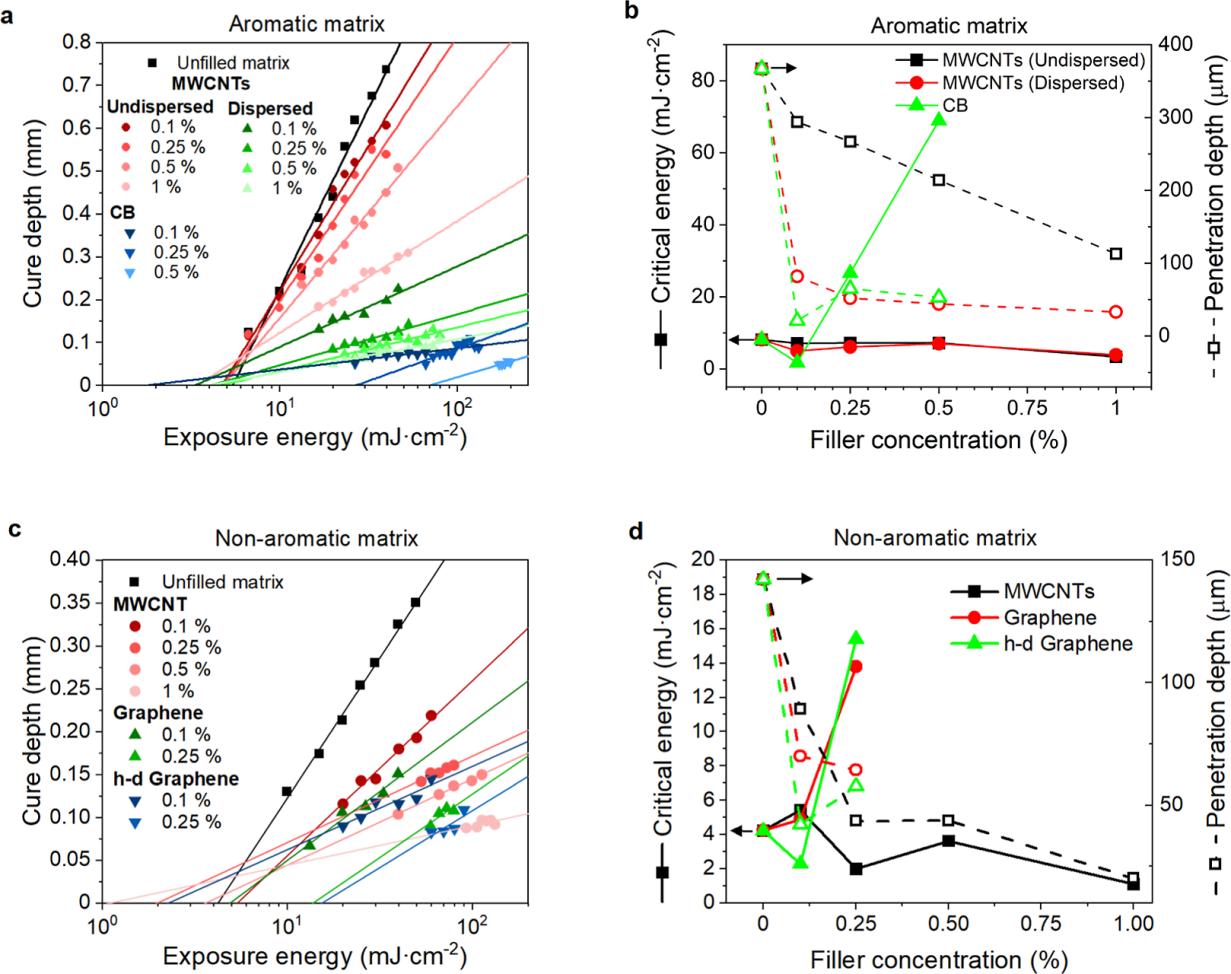
(a,c) Jacobs working curves and (b,d) critical energy (solid symbols) and penetration depth (open symbols) concentration dependence for the aromatic (a,b) and nonaromatic matrix (c,d) nanocomposite resins.

**Figure 4:**
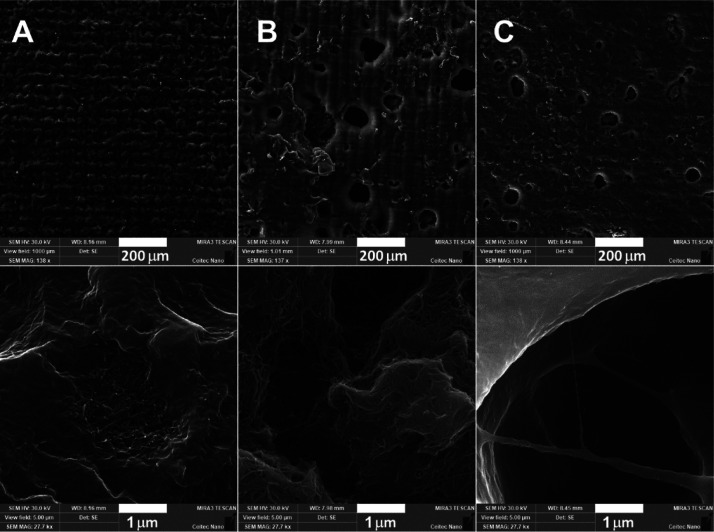
SEM micrographs of 0.25% of CNTs in (a) aromatic (dispersed), (b) aromatic (undispersed), and (c) nonaromatic matrices at low (top) and high (bottom) magnifications.

Incorporating nanofillers into a photopolymer resin may alter its curing properties.^28^ The Jacobs working curves for the CB and MWCNTs in the aromatic matrix are depicted in Figure 3a. The calculated values of critical energy (*E*_c_) and penetration depth (*D*_p_) are plotted in Figure 3b and listed in Table S8. A higher amount of MWCNTs decreases the *D*_p_ due to the filler’s light absorption characteristics and reduces *E*_c_,_,_ as expected from a polymer-adsorbing nanofiller.^28^ Consequently, the exposure time required for curing a 50 μm layer is prolonged (Table S8). Nonetheless, the determined values are strongly affected by the filler dispersion. Mixtures prepared by mechanical mixing alone contained transparent spots with randomly distributed black areas of the agglomerated filler (Figure S7). A detailed image of these inhomogeneities was collected by SEM (Figure 4b). Ultrasonication improved the homogeneity and filler dispersion (Figure 4a) and changed the sample’s optical clarity from semitransparent to homogeneously black already at 0.1% of MWCNTs (Figure S7), further decreasing the *D*_p_. At the same time, the effect on *E*_c_ was less pronounced (Figure 3b and Table S8). Penetration depth is an essential technological parameter determining the detail quality or the layer thickness achievable with a given 3D printing resin. A possible way of countering the low effective irradiation intensity caused by the light-absorbing fillers is to develop novel, highly efficient photoinitiating systems.^47^

Moreover, CB had a more significant adverse impact on the curing process than MWCNTs. While MWCNTs mainly reduced the *E*_c_, it was coarsely increased above 0.25% CB (Table S8). Although a low *E*_c_ (1.72 mJ·cm^–2^) was determined at 0.1% CB, the printability was relatively poor due to the exceptionally low *D*_p_ of 21 μm, which prolonged the layer curing time and limited the layer thickness. On the other hand, the sample containing 1% CB could not be cured even at the maximum tested exposure energy (198.3 mJ·cm^–2^). The exposure times for the 3D printing were chosen based on the Jacobs working curves. All aromatic resin samples containing MWCNTs and the 0.1% CB sample were printed with the first/other layer exposure of 120/30 s. The first and regular layer exposure times were 200 and 100 s for the sample containing 0.25% of CB, respectively. The 0.5 and 1% CB samples were not printed because of their poor photocurability.

As demonstrated above, the nonaromatic matrix struggles to keep good compatibility with the hydrophobic carbonaceous fillers due to the lack of strongly interacting sp^2^ hybridized aromatic cycles. SEM images revealed only a few individual nanotubes near larger threads (Figure 4c). Similar bundles were observed in the neat MWCNTs (Figure S3). This semidispersed state correlates well with the rheological (Figure 1) and dielectric properties (Figure 2), suggesting that the carbonaceous fillers cannot be fully dispersed in the nonaromatic matrix.

Jacobs working curves for the MWCNTs, graphene, and h-d graphene in the nonaromatic matrix are shown in Figure 3c. The *E*_c_ and *D*_p_ values are plotted in Figure 3d and listed in Table S6. Although the nonaromatic matrix had a significantly shorter *D*_p_ (142 μm, Figure 3d) than the aromatic one (367 μm, Figure 3b), MWCNTs reduced it to a similar level. For instance, *D*_p_ was 82 and 89 μm for 0.1% of MWCNTs in aromatic and nonaromatic resins, respectively. On the other hand, the *E*_c_ values scaled more proportionally to the unfilled resins (aromatic/nonaromatic: 8.14/4.22 mJ·cm^–1^, Figure 3b,d). Graphene had a more pronounced adverse impact on the curing properties than MWCNTs (Figure 3d and Table S8). Therefore, the exposure time was set to 120/30 s for the first/other layers, and the print layer thickness was shortened from 50 to 25 μm to ease the printing of highly loaded samples. 0.1 and 0.25% of MWCNTs were printed in the same setting for better reference. However, the prints with 0.5 and 1% MWCNTs failed, plucking the printed object from the platform just after curing a few layers. It was probably caused by the combination of high viscosity and poor homogeneity, despite the MWCNTs-filled aromatic resins being printed at 0.5% loading at a similar viscosity (≈10 Pa·s).

Once the prepared formulations’ printability, viscosity, and dispersion stability were defined, FTIR spectroscopy (Figure S4a,d) was used to identify the curing efficiency from the signal change in the C=C region (Figure S4b,e). The aromatic matrix featured a peak of reactive C=C bonds at around 1648 cm^–1^, contributing 19.06% of the total C=C signal (Figure S4c).^28^ It was accompanied by two triplets assigned to nonreactive aromatic cycles (Figure S4c).^33^ Therefore, the curing efficiency of the aromatic matrix formulations was calculated from the peak found at 1648–1650 cm^–1^ after deconvolution (Table S4). This method is much less precise than the direct determination of the C=C signal change in the nonaromatic matrix (Table S4) and may lead to pronounced errors in the conversions.

Figure S4e displays a region typical for the acrylic C=C bond of the nonaromatic resin before and after 3D printing. The peak intensity diminished, corresponding to 88.6 and 77.6% conversion for the upper (first layer) and bottom (last layer) 3D printed sides, respectively. The upper layer’s higher conversion is caused by the longer first-layer illumination and, potentially, by receiving an additional dose when the second (and other) layer is cured. Table S4 reports the calculated conversion of the printed specimens. Carbonaceous fillers worsened the double bond conversion due to intense light absorption (Table S4).^43^ The FTIR–ATR spectroscopy is a surface-specific technique, and a slightly different curing profile might be expected in bulk.

### 3.4 Thermomechanical Properties of Printed Specimens

**Figure 5:**
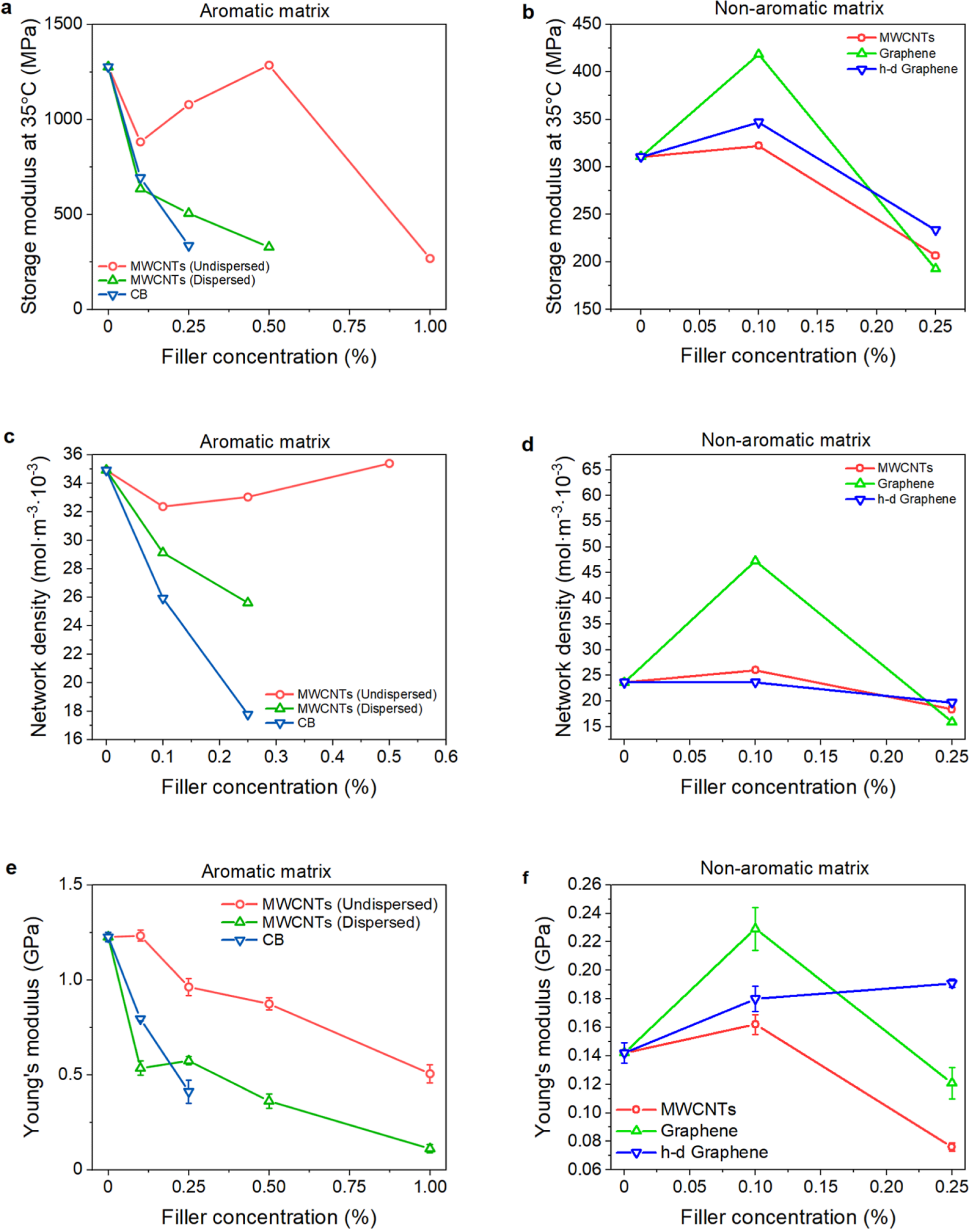
(a,b) Initial storage modulus (at 35 °C), (c,d) network density, and (e,f) Young’s modulus as a function of the filler loading in (a,c,e) aromatic and (b,d,f) nonaromatic matrix nanocomposites.

3D-printed specimens were tested for mechanical and thermomechanical properties. The investigated concentration range was limited to 0.5% of the dispersed MWCNTs and CB in the aromatic matrix due to the poor printability of highly filled resins caused by their high viscosity (Figure 1a) and slow curing (Figure 3a,b). Figures 5a–d and S8a–d present the results of the hybrid DMA–HDT analysis.^34^ The final photopolymer properties are tied to its curing degree.^34^ The worsened mechanical performance recorded for aromatic matrix nanocomposites (Figures 5a and S8a,c) corresponded to the reduced network density (Figure 5c) due to the deteriorated conversion (Table S4). These results correlated with Young’s modulus (Figure 5e) and ultimate strength (Figure S8e) established by the tensional test. The same moduli of the sonicated CB and MWCNT samples (Figure 5e) compared to the CB samples’ higher network density (Figure 5c) document the reinforcing capability of the MWCNT networks. Undispersed MWCNTs had a less significant impact on the mechanical performance because of their better conversion. This conclusion is somewhat unintuitive since enhancing thermomechanical properties usually favors a good nanofiller dispersion.^42^ Interestingly, their percolation threshold above 0.5% was apparent only in the DMA–HDT data (Figure 5a) but not in the tensile test (Figure 5e). It suggests that the percolation could be only detected in shear or compression, which inherently contributes to the bending deformation mode used in the DMA–HDT measurement. Pursuing this issue is worth further investigation since it may reveal new facts about joining layers in VPP techniques.

Thermomechanical properties of the nonaromatic matrix composites are displayed in Figure 5b,d. All fillers increased the initial storage modulus at 35 °C at a 0.1% content (Figure 5b). The most remarkable improvement of 35% was observed for the graphene-loaded samples. Similarly, the increase of network density and HDT was observed in all samples at 0.1% filler content (Figures 5d and S8b), and only graphene exhibited a lowered *T*_g_ (Figure S8d). However, deteriorated performance was obtained at higher filler contents due to the worsened conversion (Table S4). The modulus increase at 0.1% filler content was also observed in the tensile test results (Figure 5f). The highest value of 0.23 GPa belonged to a nanocomposite containing 0.1% of graphene. However, increasing the MWCNTs and graphene content decreased the elastic modulus due to poor curing. The filling also worsened the ultimate strength except for 0.1% of graphene (Figure S8f).

### 3.5 Dielectric and Electrical Properties of 3D-Printed Materials

**Figure 6:**
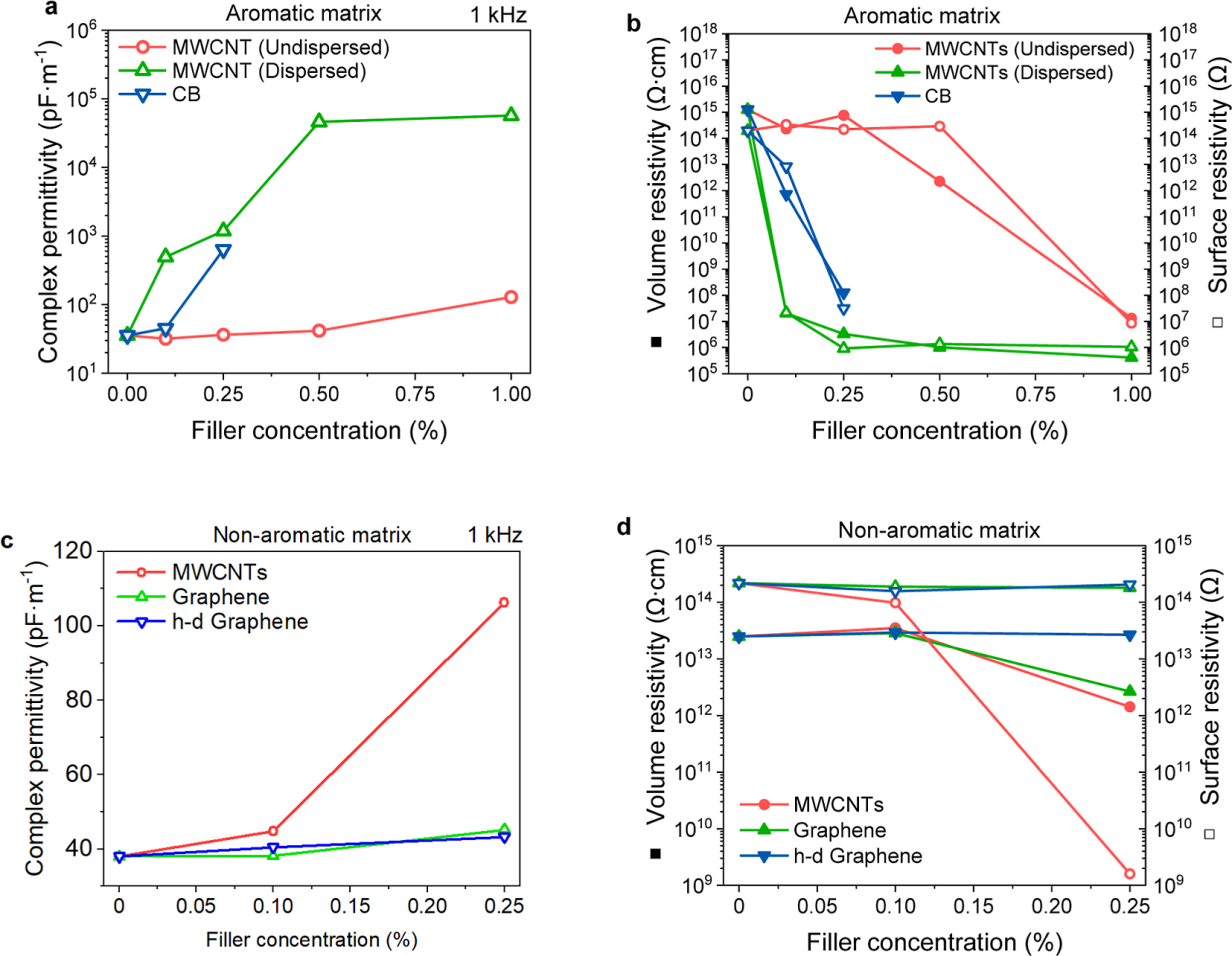
(a,c) Complex permittivity at 1 kHz and (b,d) volume (solid symbols) and surface resistivity (open symbols) of aromatic (a,b) and nonaromatic (c,d) matrix composites as a function of the filler loading.

Figure 6a shows the complex permittivity of the printed aromatic matrix specimens at 1 kHz as a function of filler loading. The curing-induced transition from a liquid to a solid restricts the mobility of charged species. It caused the complex permittivity to drop by approximately 2–3 orders of magnitude relative to that of uncured resin. Inhomogeneity in the samples, imperfect layer connection, and the absence of an efficient conductive network may further reduce the permittivity.^11^ A good filler dispersion is beneficial for high permittivities also after curing/printing. For instance, 1% of MWCNTs increase the permittivity nearly 450 times more when dispersed (57 nF·m^–1^). The undispersed samples (128 pF·m^–1^) were already outperformed by 0.1% of the same filler when dispersed (494 pF·m^–1^). Analogically, the AC conductivity at 1 kHz increased from 8.06 × 10^–11^ S·cm^–1^ for the pristine matrix to 3.58 × 10^–6^ and 3.81 × 10^–8^ S·cm^–1^ for 1% CB and dispersed MWCNTs, respectively (Table S7). MWCNTs represent a more efficient filler for adjusting electric properties than CB. The threshold where the insulating matrix turns into a semiconductor^48^ was observed at 0.5% of dispersed MWCNTs. The DC electrical resistivity values are plotted in Figure 6b. Considering both measurements, the dielectric and electrical percolation thresholds for the undispersed MWCNTs lie around 0.5% and are shifted to 0.1% by ultrasonication-enhanced dispersion. The CB’s percolation threshold assumingly appears between 0.1 and 0.25%, but the percolated samples are hardly processed due to their poor curing properties.

The initial permittivity values of the uncured nonaromatic formulation (∼10^2^ pF·m^–1^) were much lower than in the aromatic resin (∼10^4^ pF·m^–1^ at 1 kHz) due to the lack of polarizable species with sp^2^ hybridization. Nonetheless, the cured resins had almost identical permittivities (35.4 and 38.0 pF·m^–1^ for aromatic and nonaromatic resin, respectively, Figure 6a,c and Table S7). Almost all nonaromatic matrix composites experienced a far less pronounced drop in permittivity during the curing (Figure 6c and Table S7) than the aromatic ones. The only exception was the 0.25% MWCNTs sample, which decreased the complex permittivity by approximately 2 orders of magnitude, similar to the aromatic matrix composites. Volume and surface resistivity as a function of the filler loading is reported in Figure 6d. It is evident that carbonaceous fillers had little impact on the dielectric and electrical properties of the nonaromatic matrix. The only exception was again the 0.25% MWCNTs filling, which formed a conductive network. Nonetheless, the dielectric/electric properties of the aromatic matrix composites (Figure 6a,b) were far superior to those of this sample (Figure 6c,d).

**Figure 7:**
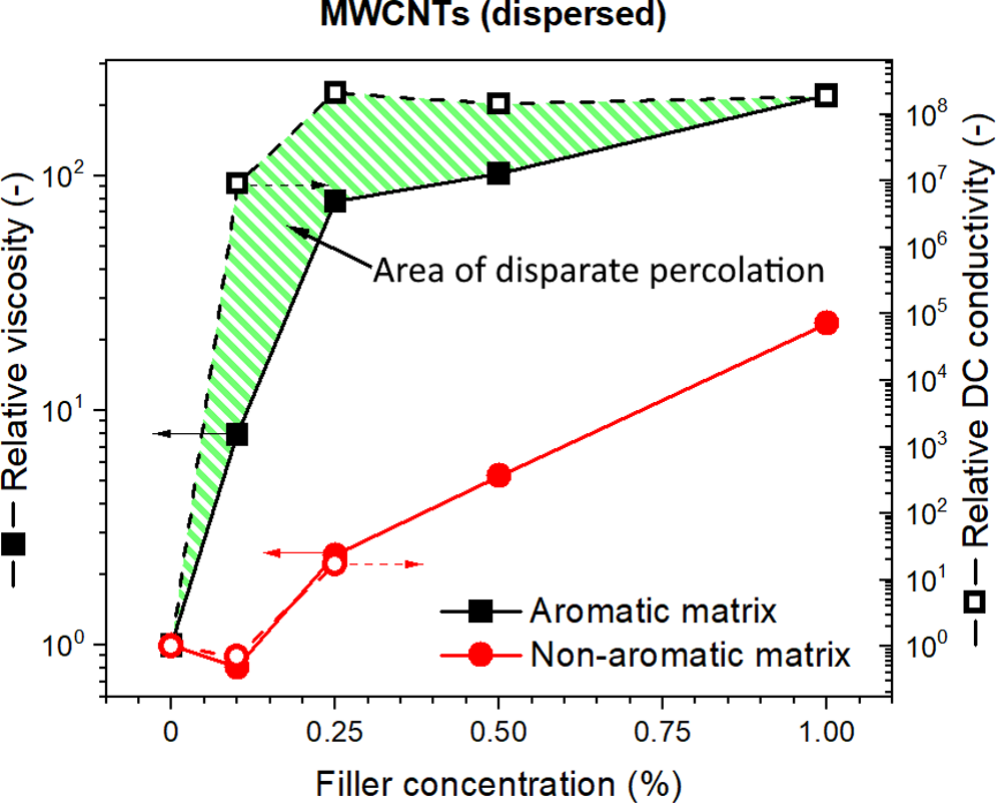
Disparate rheological and electrical percolation was obtained by comparing relative viscosity and DC conductivity. The scales on *y*-axes were adjusted such that the first and last points were aligned. This was necessary since the conductivity changed by 8 orders of magnitude while the viscosity changed only by 2.

Finally, the percolation of rheological and electrical properties for dispersed MWCNTs is compared in Figure 7. The resin viscosity (Figure 1a,b) and DC conductivity (obtained as 1/volume resistivity; Figure 6b,d) were converted into relative values and plotted against the filler concentration. The scales on the *y*-axes were adjusted such that the first and last points were aligned. This was necessary since the conductivity changed by 8 orders of magnitude while the viscosity changed only by 2. Notably, the despaired percolation area appeared only in the aromatic matrix samples. Therefore, we suggest that the high permittivity induced by the strong polymer–filler interaction is important for the tunneling mechanism, which supposedly lowered the electric percolation. Indeed, the electron tunneling rate in semiconductors is a function of the relative permittivity.^49^ Given the practical potential of this observation, the phenomenon should be further studied in more detail by varying the parameters of the sp^2^ hybridized species in the resin, mediating good interactions with the carbonaceous fillers.

## 4 Conclusions

This study compared the impact of carbonaceous fillers (CB, MWCNTs, graphene, and highly defective graphene) on the aromatic and nonaromatic photopolymer resins’ properties. The samples were tested for viscosity, long-term stability, complex permittivity, curing efficiency, final conversion, storage modulus, heat deflection and glass transition temperatures, network density, and DC resistivity. Our results conclude that the improved electric and dielectric properties are closely tied to the dispersion quality, favoring enhanced dispersion by ultrasonication. Dispersed fillers also reduced the critical energy *E*_c_ needed to cure a solid layer. On the other hand, the dispersed filler worsened the printability due to the elevated viscosity and deteriorated optical clarity and penetration depth *D*_p_. It also increased energy losses in the electric AC field. MWCNTs were found to be a more influential filler than CB for both positive and adverse effects due to their anisotropic shape and lower percolation threshold. The most extensive changes in conductivity were observed below the percolation limit, while the complex permittivity continued to increase at higher filler loadings. Nonetheless, the rheological and electrical percolation thresholds were disparate. It leaves a potentially interesting window for practical applications with enhanced electrical properties while not suffering much from increased viscosity.

Additionally, the filler–resin compatibility ensured by the resin’s aromatic character was demonstrated as a crucial factor for increasing the complex permittivity and conductivity of the printed materials. Highly defective graphene was more hydrophilic due to the increased oxygen concentration, yet it had only a limited impact on the functional properties. The nonaromatic resin reduced the filling efficiency and compromised the long-term stability by showing hints of aggregation after 14 days of storage. A drop of complex permittivity at 1 kHz by 2–3 orders of magnitude accompanied the curing except for samples with poor dielectric performance, where nearly no change was observed. This effect was caused by the reduced mobility of polarizable species in the cured material. The presented results highlight challenges that must be addressed in designing and processing carbonaceous filler-based 3D printing resins. They not only attempt to guide through the selection of the filler type and concentration and a compatible matrix but also highlight the importance of good dispersion and curing conditions.

## Data Availability

The processed data required to reproduce the above findings are available as a supplementary electronic file.

## Supporting Information Available

Filler analysis, including BET isotherms, XPS, Raman spectroscopy, and SEM analysis; FTIR spectroscopy; rheological properties; DETA; photos of 3D-printed aromatic matrix composites; curing properties; and thermomechanical properties (PDF)
