# Supplementary material for: Matching Low Viscosity with Enhanced Conductivity in Vat Photopolymerization 3D Printing: Disparity in the Electric and Rheological Percolation Thresholds of Carbon-Based Nanofillers Is Controlled by the Matrix Type and Filler Dispersion
Source: ACS Omega. 2023 Nov 25;8(48):45566–77. doi: 10.1021/acsomega.3c05683 (PMC10701886; doi:10.1021/acsomega.3c05683)
Supplement: Supplementary file 1 — ao3c05683_si_001.pdf [file ao3c05683_si_001.pdf]

Matching low viscosity with enhanced conductivity in vat photopolymerization 3D printing: Disparity in electric and rheological percolation threshold of carbon-based nanofillers is controlled by matrix type and filler dispersion

Supplementary information

Veronika Sevriugina[1], David Pavliňák[1], František Ondreáš[1,2], Ondřej Jašek[3], Martina Štaffová[1], Petr Lepcio[1*]

[1] Central European Institute of Technology, Brno University of Technology, Purkyňova 123a, 612 00, Brno, Czech Republic

[2] Contipro a.s., Dolni Dobrouc 401, 56102 Dolni Dobrouc, Czech Republic

[3] Department of Physical Electronics, Faculty of Science, Masaryk University, Kotlářská 267/2, 611 37 Brno, Czech Republic

*petr.lepcio@ceitec.vutbr.cz

1. Filler analysis

Brunauer–Emmett–Teller (BET) isotherm

Sample	BET surface area (m^2/g)
MWCNT	258
CB	973
Graphene	160
h-d Graphene	231

Tab. S1: BET analysis of carbonaceous fillers

X-ray photoelectron spectroscopy (XPS)

XPS (at.%)						
Sample	From wide spectrum			From Hi-res spectra		
	C	O	N	C	O	N
MWCNT	98.9 +/-0.4	1.1 +/-0.4	0	99.3 +/-0.1	0.7 +/-0.1	0
CB	99.3 +/-0.3	0.7 +/-0.3	0	99.1 +/-0.4	0.9+/-0.4	0
Graphene	99.1 +/-0.7	0.9 +/-0.7	0	98.8 +/-0.4	1.2 +/-0.4	0
h-d Graphene	98.1 +/-0.1	1.9 +/-0.1	0	97.9 +/-0.3	2.1 +/-0.3	0

EDX/SEM (wt.%)				
	C	O	N	Al
MWCNT	92.1	4.4	-	3.5
CB				
Graphene	97.8	2.2	-	-
h-d Graphene	99.1	0.88	-	-

Tab. S2: Elemental analysis by XPS and EDX(SEM) for carbonaceous fillers

at.%				
Sample	C-C	Pi-Pi	CO	C=O
MWCNT	83.1	11.0	3.7	2.3
CB	89.3	7.1	1.7	1.9
Graphene	87.1	8.2	3.4	1.3
h-d Graphene	88.0	6.1	3.0	3.0

Tab. S3: XPS Hi-Res analysis of C1s for carbonaceous fillers

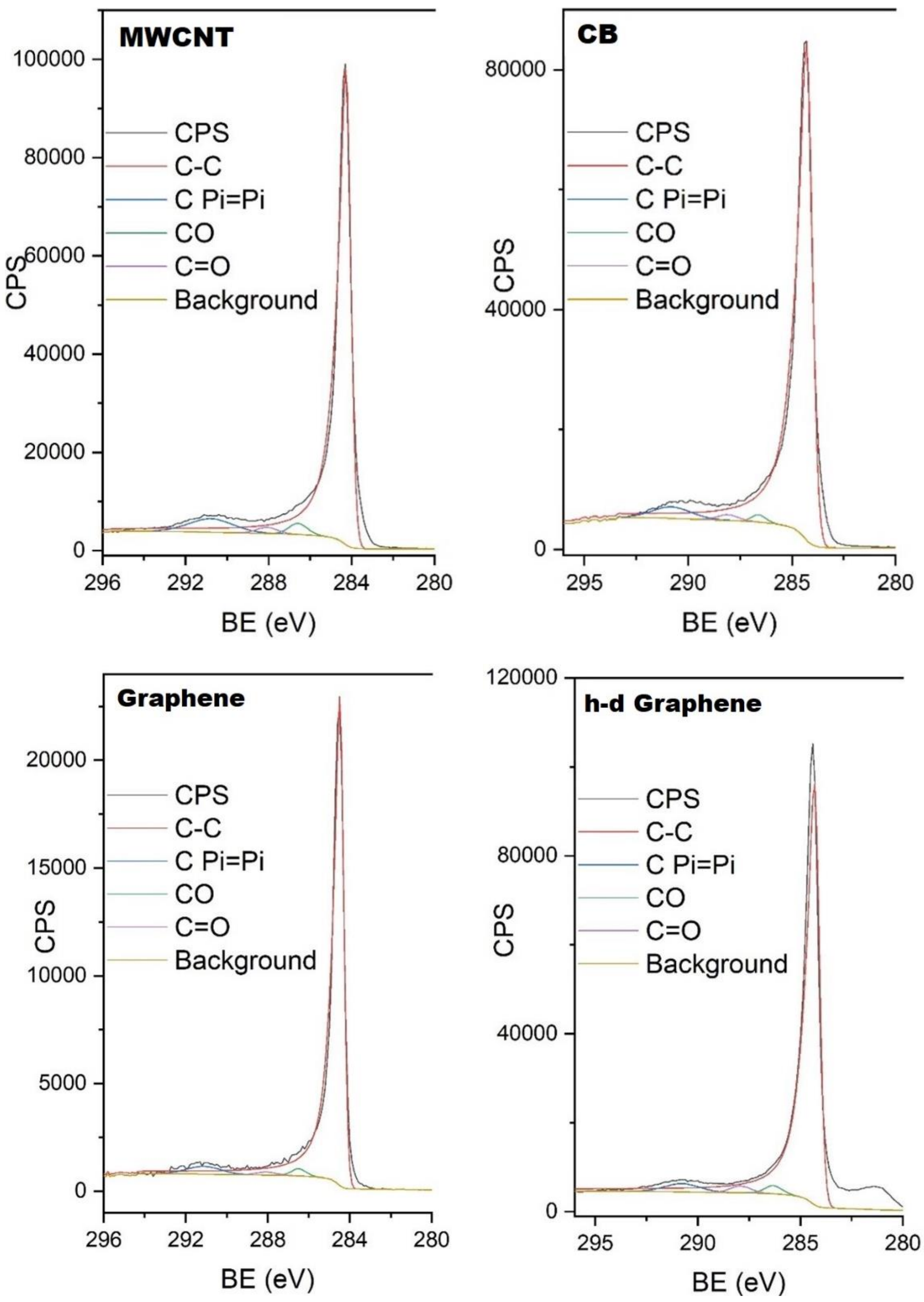

Fig. S1: Hi-Res XPS spectra C1s of carbonaceous fillers

Raman spectroscopy

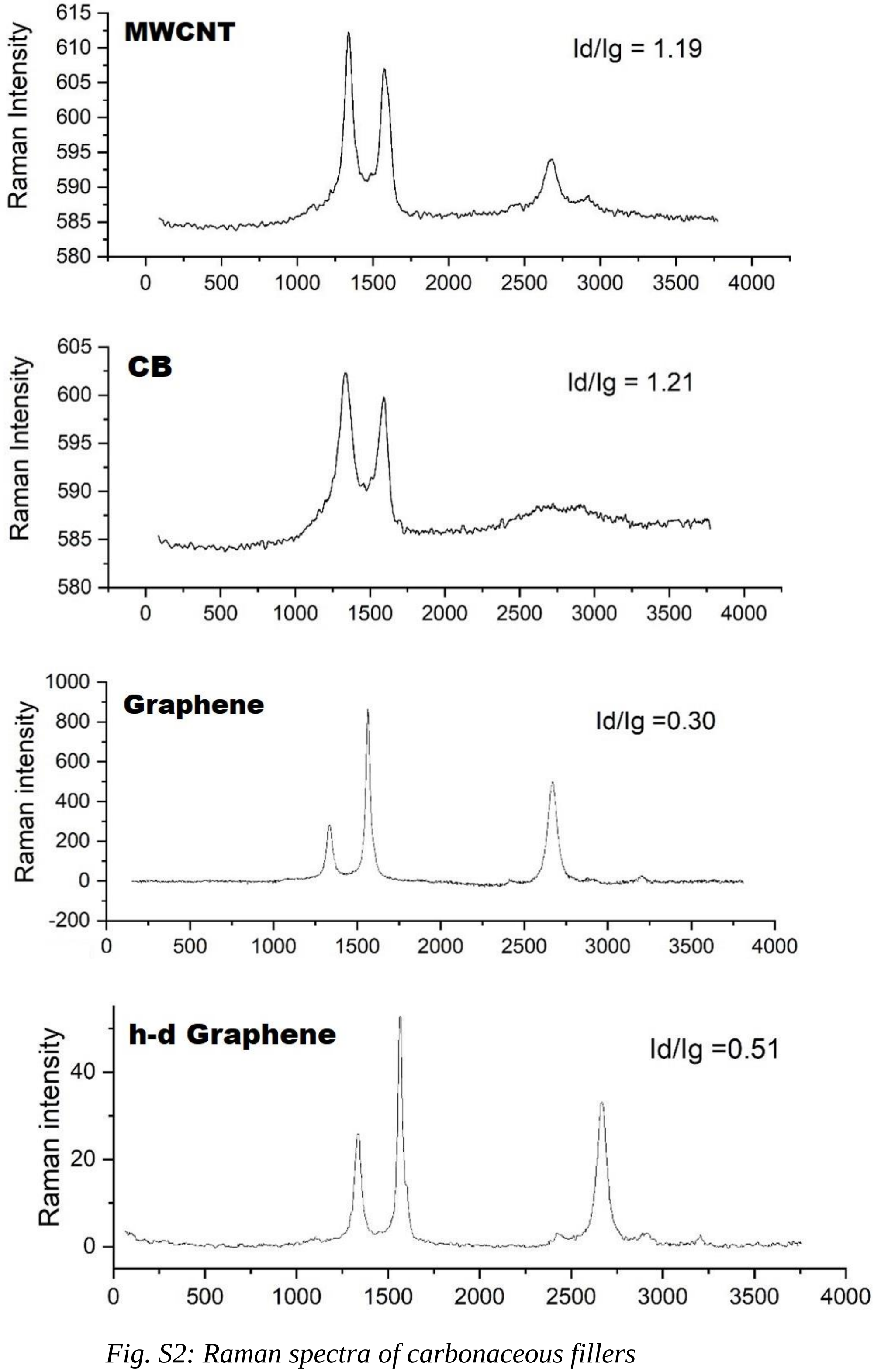

Fig. S2: Raman spectra of carbonaceous fillers

SEM analysis

MWCNT

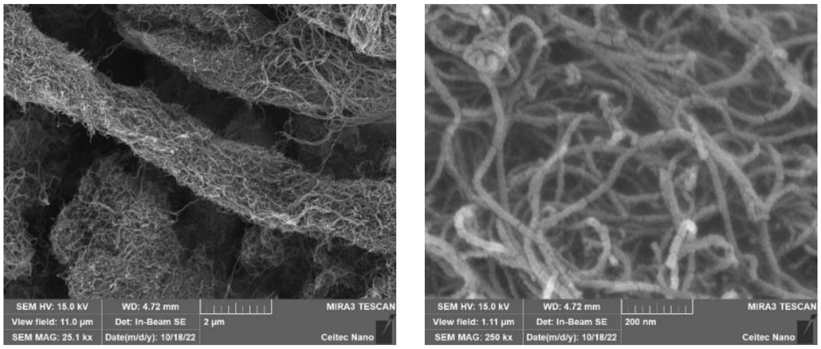

CB

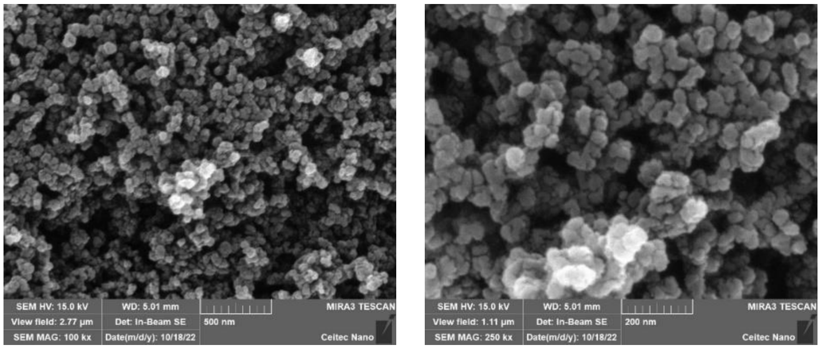

Graphene

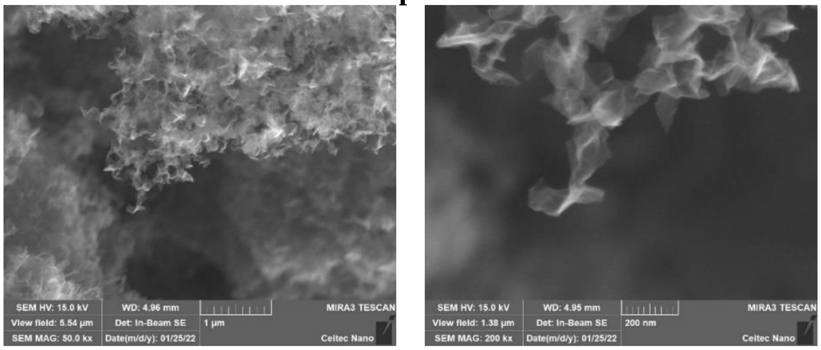

h-d Graphene

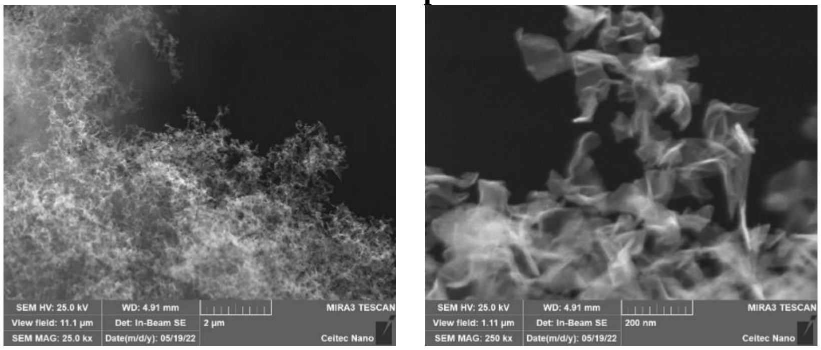

Fig. S3: SEM images of carbonaceous fillers

2. FTIR spectroscopy

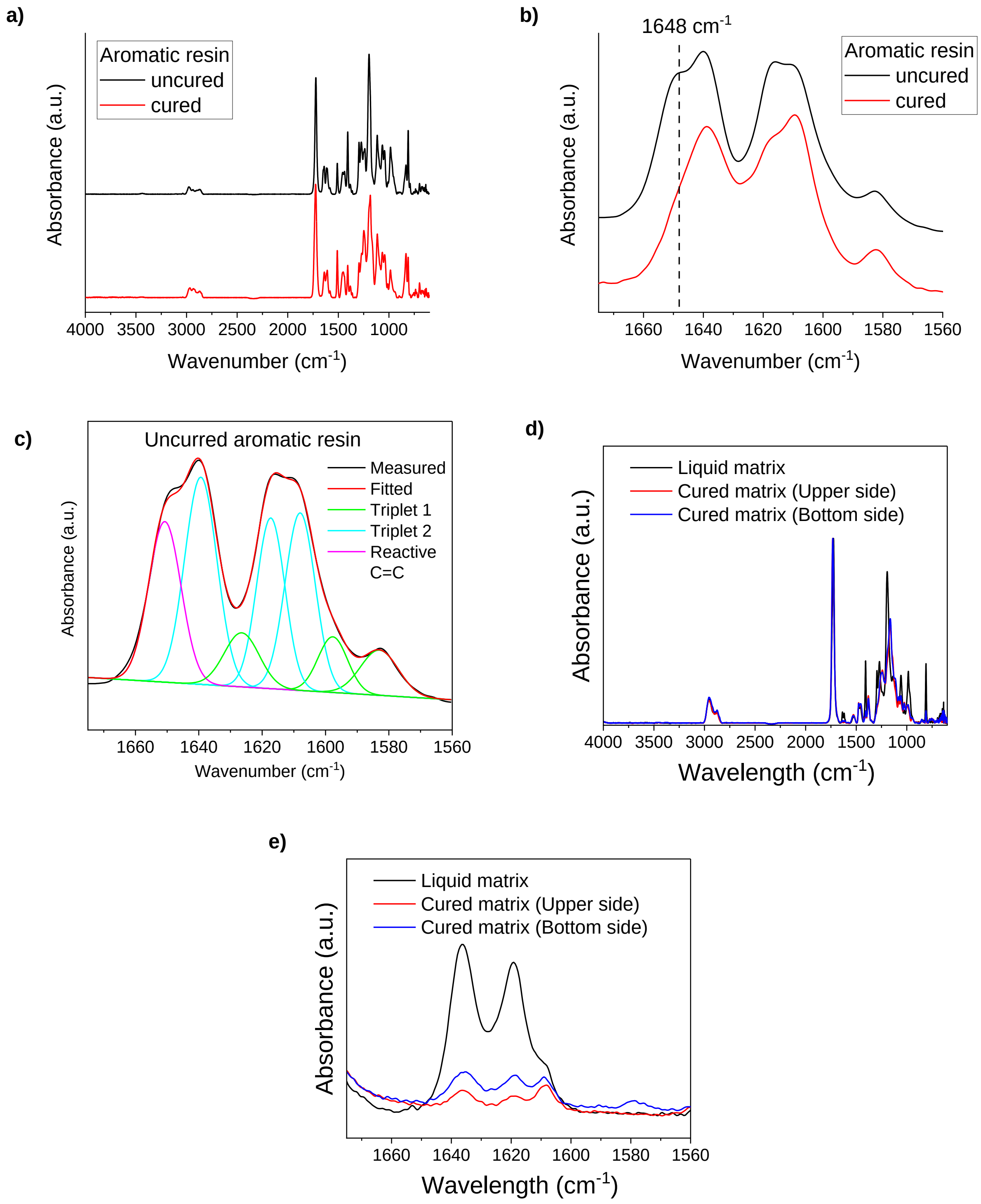

Fig. S4: (a) FTIR spectra, and (b) detail of the C=C signal of the cured (red) and uncured (black) aromatic resin. (c) deconvolution of the uncured C=C signal. (d) FTIR spectra, and (e) detail of the C=C signal of the uncured (black) and cured upper (red) and bottom (blue) side of non-aromatic resin

	Type of NPs	Concentration (%)	Conversion (%)	
Aromatic matrix	Unfilled matrix	-	54.50	
	MWCNTs (undispersed)	0.1	30.79	
		0.25	47.95	
		0.5	46.83	
		1	45.16	
	MWCNTs (dispersed)	0.1	49.67	
		0.25	33.05	
		0.5	47.05	
		1	38.84	
			**Upper side**	**Bottom side**
	CB	0.1	61.98	42.24
		0.25	56.78	38.66
		0.5	46.87	25.34
			**Upper side**	**Bottom side**
Non-aromatic matrix	Unfilled matrix	-	88.58	77.61
	MWCNTs	0.1	91.16	79.78
		0.25	74.22	68.33
	Graphene	0.1	88.60	64.46
		0.25	74.37	76.90
	h-d Graphene	0.1	79.10	80.34
		0.25	74.33	77.74

Tab. S4: Conversions of aromatic and non-aromatic matrix nanocomposites after printing.

3. Rheological properties

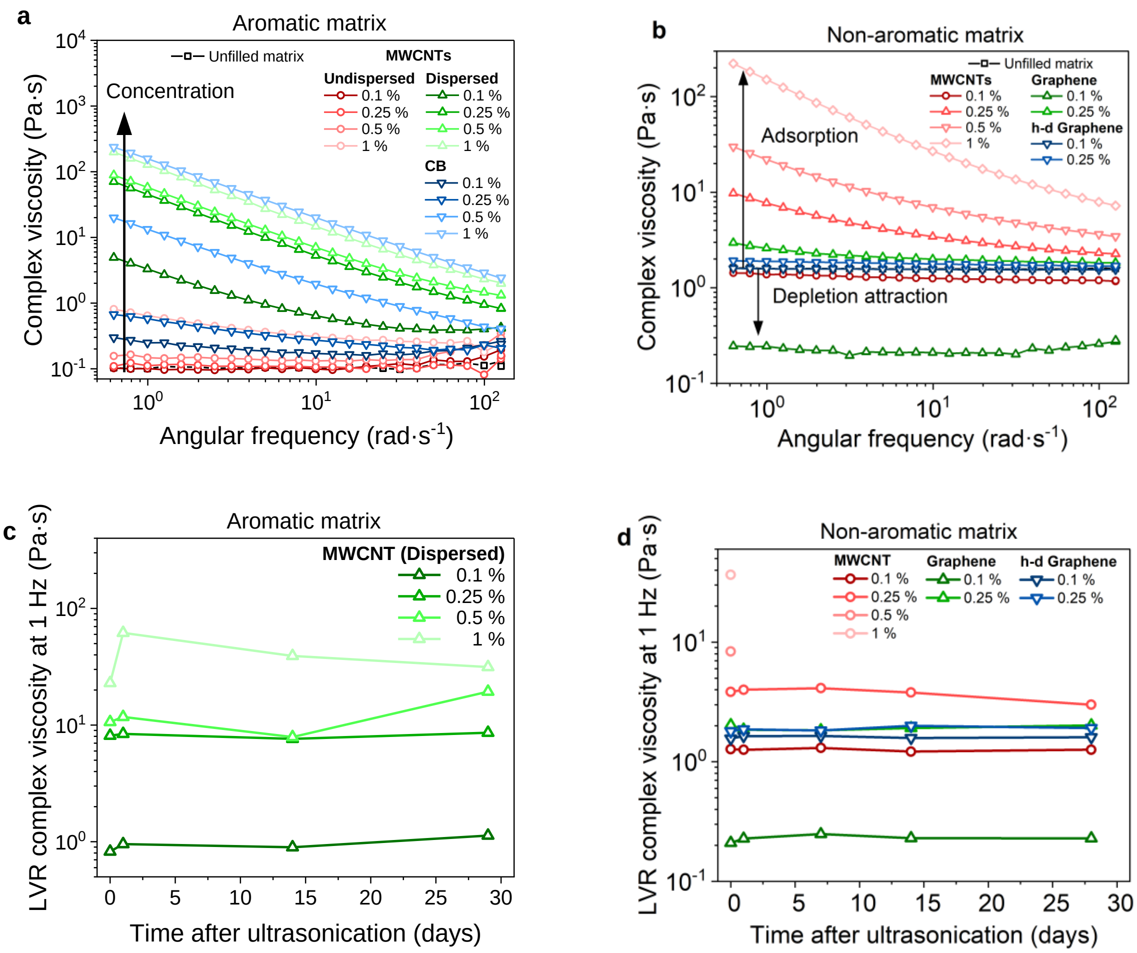

Fig. S5: (a,b) Complex viscosity as a function of angular frequency, and (c,d) linear viscoelastic range (LVR) complex viscosity at 1 Hz (η_{1Hz}) as a function of the time after ultrasonication for (a,c) aromatic and (b,d) non-aromatic matrix nanocomposite resins.

Matrix	Type of NPs	Concentration (%)	η_{1Hz} [Pa·s]	n
Aromatic matrix	Unfilled resin	-	0.104	0.988
	MWCNTs (Undispersed)	0.1	0.100	1.010
		0.25	0.106	0.990
		0.5	0.135	0.939
		1	0.341	0.673
	MWCNTs (Dispersed)	0.1	0.823	0.205
		0.25	8.114	0.059
		0.5	10.640	0.078
		1	22.932	0.047
	CB	0.1	0.180	0.855
		0.25	0.303	0.636
		0.5	2.754	0.125
		1	30.736	0.111
Non-aromatic matrix	Unfilled resin	-	1.584	0.993
	MWCNTs	0.1	1.276	0.960
		0.25	3.839	0.596
		0.5	8.361	0.437
		1	36.633	0.211
	Graphene	0.1	0.210	0.976
		0.25	2.025	0.832
	h-d Graphene	0.1	1.563	0.991
		0.25	1.797	0.970

Tab. S5: Complex viscosity at 1 Hz linear viscoelastic region (LVR) η_{1Hz}, and power law index n for freshly prepared uncured aromatic and non-aromatic matrix nanocomposite resins.

4. Dielectric thermal analysis (DETA)

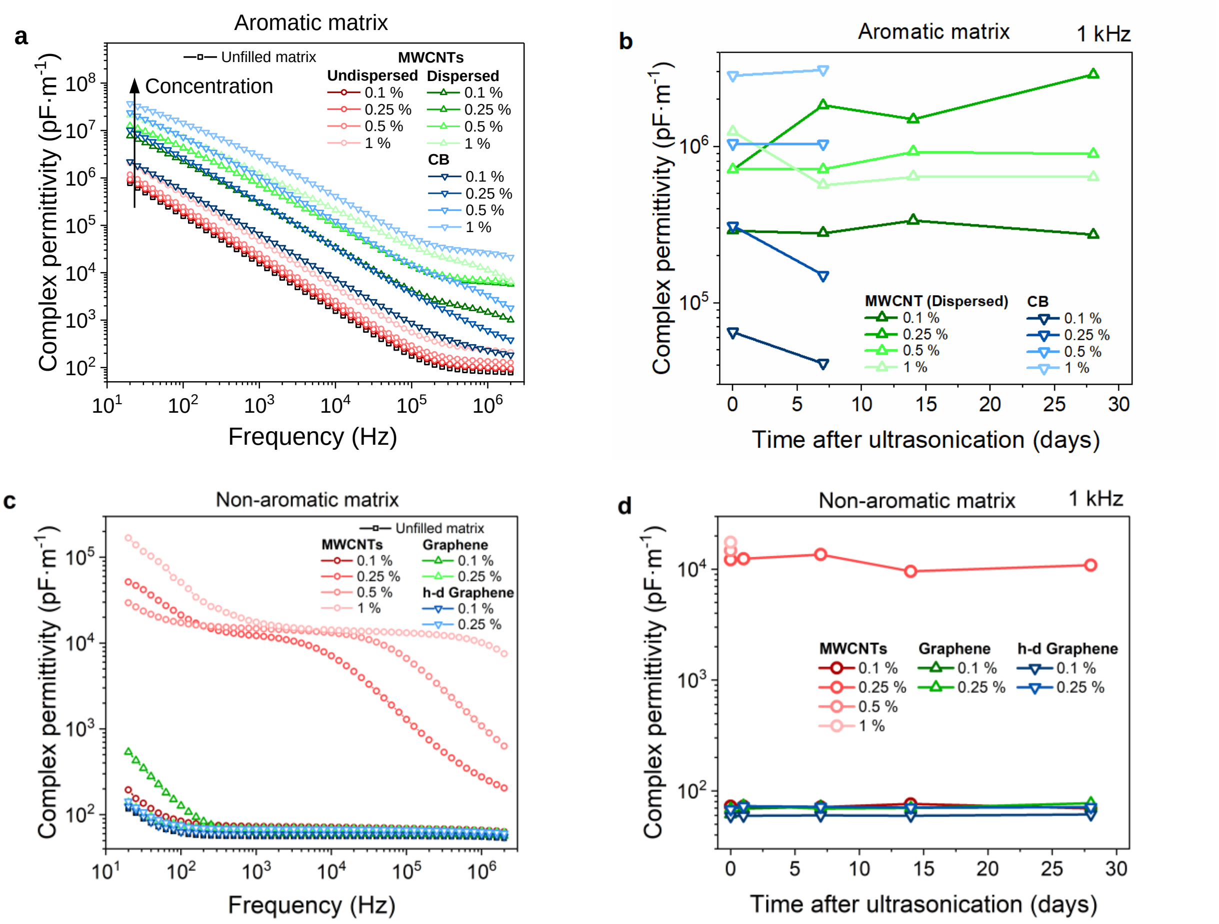

Fig. S6: Complex permittivity as a function of (a,c) frequency and (b,d) time after ultrasonication (at 1 kHz) for the uncured (a,b) aromatic and (c,d) non-aromatic matrix nanocomposite resins.

Matrix	Filler	Concentration [%]	ε^* [nF·m^{-1}]	σ [S·cm^{-1}]	tanδ [-]
Aromatic matrix (uncured)	Unfilled resin	-	15.57	$9.80\cdot10^{-7}$	153.60
	MWCNTs (Undispersed)	0.1	18.10	$1.14\cdot10^{-6}$	117.52
		0.25	19.81	$1.25\cdot10^{-6}$	71.42
		0.5	25.06	$1.58\cdot10^{-6}$	96.79
		1	46.76	$2.94\cdot10^{-6}$	39.14
	MWCNTs (Dispersed)	0.1	290.55	$1.81\cdot10^{-5}$	7.15
		0.25	712.14	$4.30\cdot10^{-5}$	3.41
		0.5	713.21	$4.30\cdot10^{-5}$	3.30
		1	1247.30	$7.22\cdot10^{-5}$	2.34
	CB	0.1	65.11	$4.10\cdot10^{-6}$	28.69
		0.25	311.08	$1.95\cdot10^{-5}$	16.51
		0.5	1043.20	$6.47\cdot10^{-5}$	5.60
		1	2840.40	$1.66\cdot10^{-4}$	2.47
Non-aromatic matrix (uncured)	Unfilled resin	-	55.66	$1.46\cdot10^{-10}$	0.04
	MWCNTs	0.1	72.70	$3.28\cdot10^{-10}$	0.07
		0.25	$1.22\cdot10^{4}$	$2.91\cdot10^{-7}$	0.41
		0.5	$1.48\cdot10^{4}$	$1.24\cdot10^{-7}$	0.13
		1	$1.75\cdot10^{4}$	$4.55\cdot10^{-7}$	0.45
	Graphene	0.1	61.43	$7.56\cdot10^{-10}$	0.20
		0.25	69.13	$1.88\cdot10^{-10}$	0.04
	h-d Graphene	0.1	59.21	$1.67\cdot10^{-10}$	0.04
		0.25	68.15	$2.05\cdot10^{-10}$	0.05

Tab. S6: Complex permittivity ε^*, AC conductivity σ, and loss tangent tanδ of uncured aromatic and non-aromatic matrix resins at 1 kHz.

Matrix	Type of NPs	Concentration [%]	ε^* [$pF \cdot m^{-1}$]	σ [$S \cdot cm^{-1}$]	$\tan\delta$ [-]
Aromatic matrix (printed)	Unfilled matrix	-	35.35	$8.06 \cdot 10^{-11}$	0.04
	MWCNTs (Not dispersed)	0.1	31.65	$5.32 \cdot 10^{-11}$	0.03
		0.25	36.14	$8.13 \cdot 10^{-11}$	0.04
		0.5	41.50	$1.17 \cdot 10^{-10}$	0.04
		1	128.26	$8.28 \cdot 10^{-10}$	0.10
	MWCNTs (Dispersed)	0.1	493.56	$1.80 \cdot 10^{-8}$	0.71
		0.25	1186.4	$6.14 \cdot 10^{-8}$	1.45
		0.5	46060	$2.90 \cdot 10^{-6}$	71.78
		1	56958	$3.58 \cdot 10^{-6}$	14.53
	CB	0.1	44.83	$1.53 \cdot 10^{-10}$	0.05
		0.25	636.04	$3.81 \cdot 10^{-8}$	3.11
Non-aromatic matrix (printed)	Unfilled matrix	-	37.98	$7.76 \cdot 10^{-11}$	0.03
	MWCNTs	0.1	44.79	$1.05 \cdot 10^{-10}$	0.04
		0.25	106.23	$1.25 \cdot 10^{-9}$	0.19
	Graphene	0.1	38.12	$7.70 \cdot 10^{-11}$	0.03
		0.25	45.18	$9.18 \cdot 10^{-11}$	0.03
	h-d Graphene	0.1	40.50	$7.76 \cdot 10^{-11}$	0.03
		0.25	43.29	$8.81 \cdot 10^{-11}$	0.03

Tab. S7: Complex permittivity ε^*, AC conductivity σ, and loss tangent $\tan\delta$ of 3D printed aromatic and non-aromatic matrix nanocomposites at 1 kHz.

5. Photos

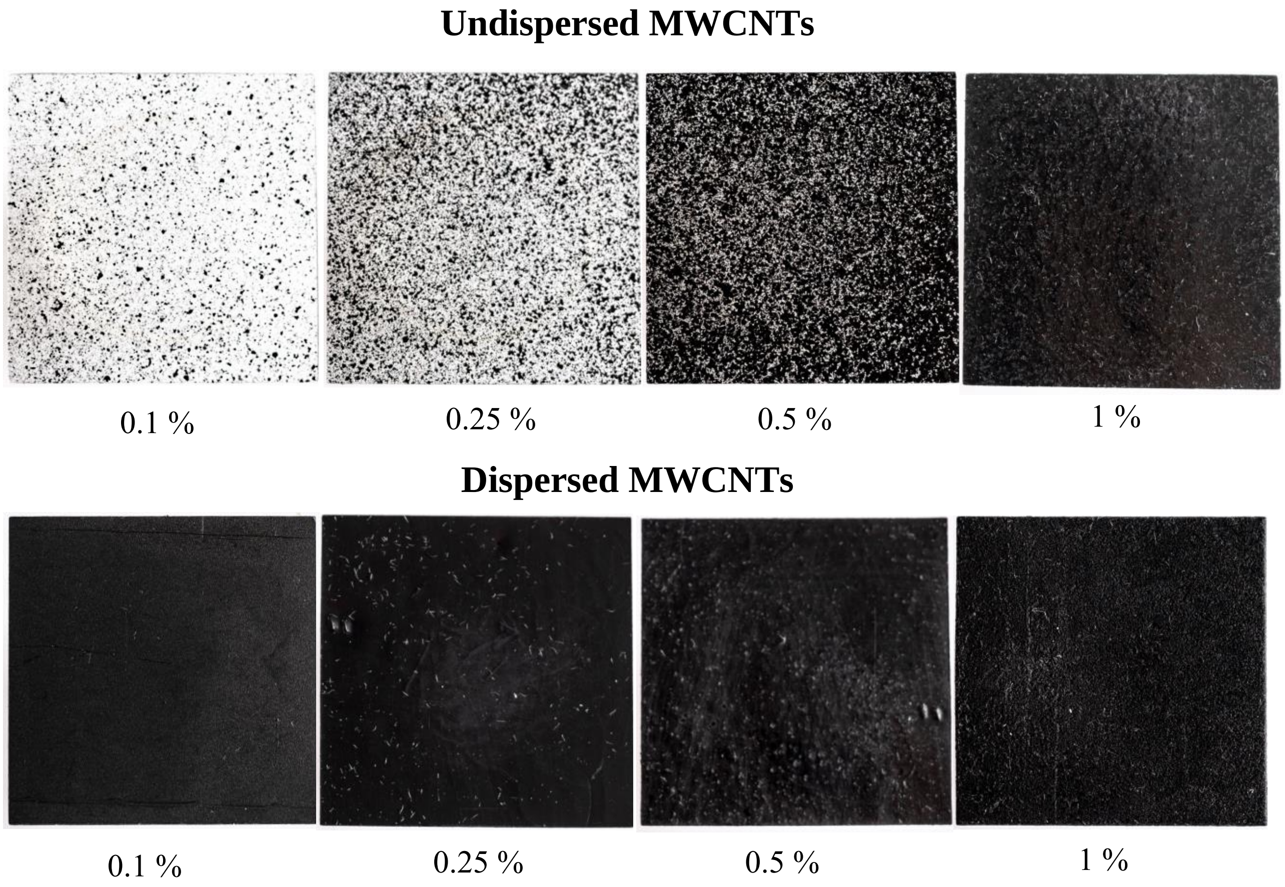

Fig. S7: Photos of 3D printed aromatic-matrix composites 40×40×1 mm.

6. Curing properties

	Filler	Concentration [%]	E_c [mJ·cm^{-2}]	D_p [µm]	$t_{Cd\text{-}50\ \mu m}$ [s]
Aromatic matrix	Unfilled resin	-	8.14	367	6.16
	MWCNTs (Undispersed)	0.1	7.09	294	5.56
		0.25	7.20	267	5.73
		0.5	7.21	214	6.02
		1	3.36	113	5.22
	MWCNTs (Dispersed)	0.1	4.98	82	6.07
		0.25	6.07	52	12.35
		0.5	7.01	44	14.35
		1	3.88	33	17.36
	CB	0.1	1.72	21	17.77
		0.25	26.61	65	57.53
		0.5	68.94	53	176.63
		1	n/a*	n/a*	n/a*
					$t_{Cd\text{-}25\ \mu m}$ [s]
Non-aromatic matrix	Unfilled matrix	-	4.22	142.1	5.04
	MWCNTs	0.1	5.43	89.2	7.18
		0.25	1.98	43.8	3.51
		0.5	3.62	43.7	9.71
		1	1.10	20.1	5.77
	Graphene	0.1	4.90	70.0	7.01
		0.25	13.79	64.3	20.35
	h-d Graphene	0.1	2.28	42.2	4.12
		0.25	15.40	57.7	23.75

*Tab. S8. Critical energy E_c, penetration depth D_p, and exposure time to cure a 50 ($t_{Cd\text{-}50\ \mu m}$) or 25 µm layer ($t_{Cd\text{-}25\ \mu m}$) for aromatic and non-aromatic matrix nanocomposite resins calculated from the logarithmic fit of Jacobs working curves. *Data not available since the sample was not cured even at the maximum tested exposure energy (198.3 mJ·cm^{-2}).*

7. Thermomechanical properties

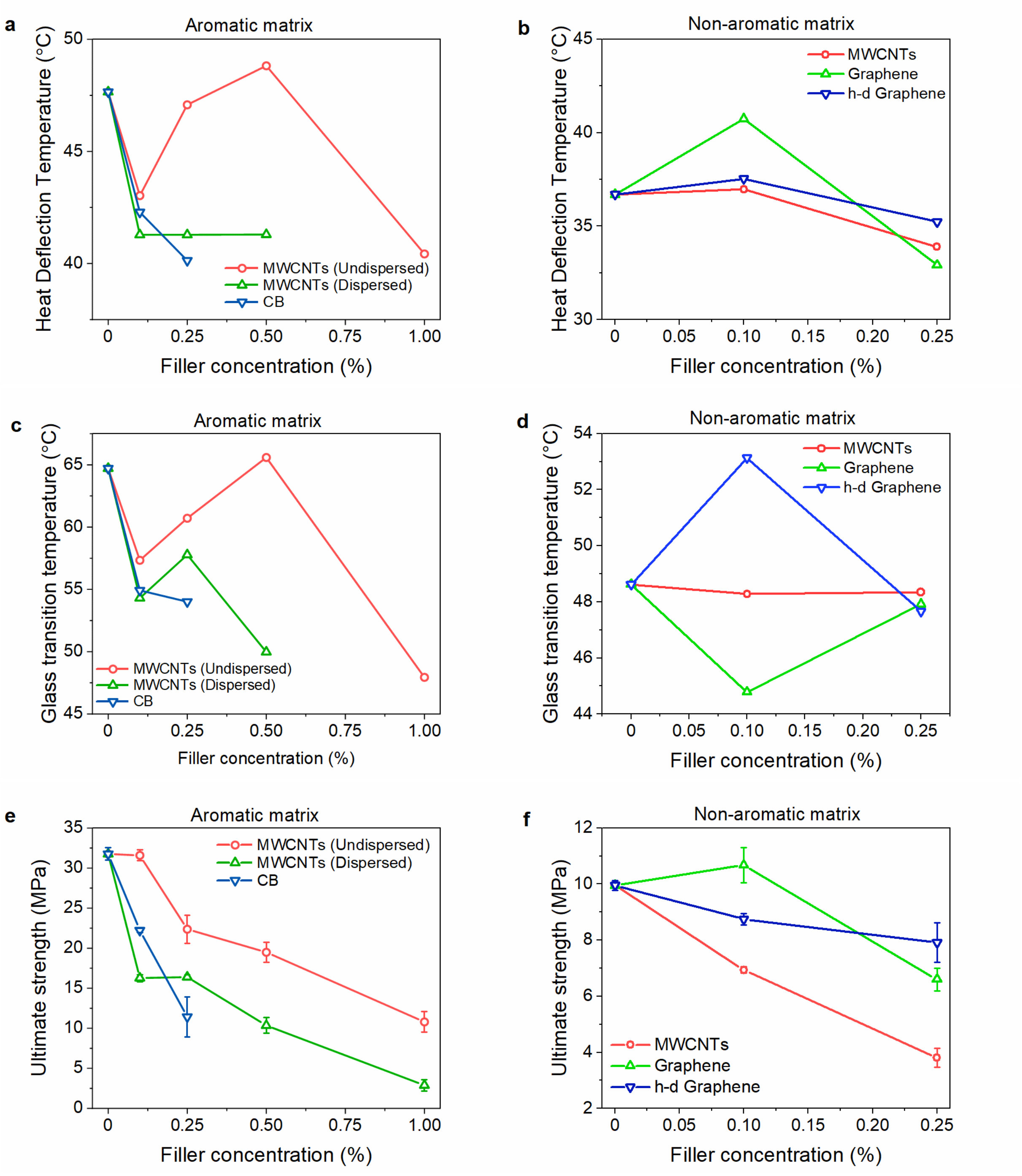

Fig. S8: (a,b) Heat deflection temperature (HDT), (c,d) glass transition temperature (T_g), and (e,f) ultimate strength as a function of filler concentration for (a,c,e) aromatic and (b,d,f) non-aromatic matrix nanocomposites.
